# Lifestyle, cardiometabolic disease, and multimorbidity in a prospective Chinese study

**DOI:** 10.1093/eurheartj/ehab413

**Published:** 2021-08-01

**Authors:** Yuting Han, Yizhen Hu, Canqing Yu, Yu Guo, Pei Pei, Ling Yang, Yiping Chen, Huaidong Du, Dianjianyi Sun, Yuanjie Pang, Ningyu Chen, Robert Clarke, Junshi Chen, Zhengming Chen, Liming Li, Jun Lv, on behalf of the China Kadoorie Biobank Collaborative Group

**Affiliations:** 1 Department of Epidemiology & Biostatistics, School of Public Health, Peking University, Xueyuan Road, Haidian District, Beijing 100191, China; 2 Peking University Center for Public Health and Epidemic Preparedness & Response, Xueyuan Road, Haidian District, Beijing 100191, China; 3 Fuwai Hospital Chinese Academy of Medical Sciences, North Lishi Road, Xicheng District, Beijing 100037, China; 4 Chinese Academy of Medical Sciences, Dongdan Santiao, Dongcheng District, Beijing 100730, China; 5 Medical Research Council Population Health Research Unit, University of Oxford, Old Road Campus, Oxford OX3 7LF, UK; 6 Clinical Trial Service Unit & Epidemiological Studies Unit (CTSU), Nuffield Department of Population Health, University of Oxford, Old Road Campus, Oxford OX3 7LF, UK; 7 NCDs Prevention and Control Department, Liuzhou CDC, Tanzhong West Road, Liunan District, Liuzhou, Guangxi 545007, China; 8 China National Center for Food Safety Risk Assessment, Guangqu Road, Chaoyang District, Beijing 100020, China; 9 Key Laboratory of Molecular Cardiovascular Sciences (Peking University), Ministry of Education, Xueyuan Road, Haidian District, Beijing 100191, China

**Keywords:** Cardiometabolic disease, Multimorbidity, Progression, Lifestyle, Prospective cohort study

## Abstract

**Aims:**

The potential difference in the impacts of lifestyle factors (LFs) on progression from healthy to first cardiometabolic disease (FCMD), subsequently to cardiometabolic multimorbidity (CMM), and further to death is unclear.

**Methods and results:**

We used data from the China Kadoorie Biobank of 461 047 adults aged 30–79 free of heart disease, stroke, and diabetes at baseline. Cardiometabolic multimorbidity was defined as the coexistence of two or three CMDs, including ischaemic heart disease (IHD), stroke, and type 2 diabetes (T2D). We used multi-state model to analyse the impacts of high-risk LFs (current smoking or quitting because of illness, current excessive alcohol drinking or quitting, poor diet, physical inactivity, and unhealthy body shape) on the progression of CMD. During a median follow-up of 11.2 years, 87 687 participants developed at least one CMD, 14 164 developed CMM, and 17 541 died afterwards. Five high-risk LFs played crucial but different roles in all transitions from healthy to FCMD, to CMM, and then to death. The hazard ratios (95% confidence intervals) per one-factor increase were 1.20 (1.19, 1.21) and 1.14 (1.11, 1.16) for transitions from healthy to FCMD, and from FCMD to CMM, and 1.21 (1.19, 1.23), 1.12 (1.10, 1.15), and 1.10 (1.06, 1.15) for mortality risk from healthy, FCMD, and CMM, respectively. When we further divided FCMDs into IHD, ischaemic stroke, haemorrhagic stroke, and T2D, we found that LFs played different roles in disease-specific transitions even within the same transition stage.

**Conclusion:**

Assuming causality exists, our findings emphasize the significance of integrating comprehensive lifestyle interventions into both health management and CMD management.

## Graphical Abstract

**Figure:**
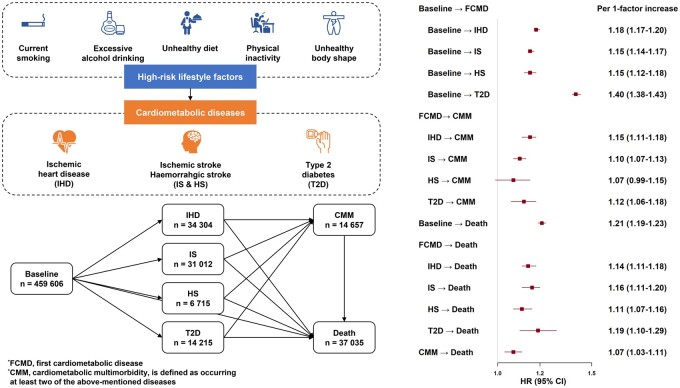
High-risk lifestyle factors are associated with all disease transition stages from healthy to first cardiometabolic disease, to cardiometabolic multimorbidity, and then to death, but to different extents.

**See page 3385 for the editorial comment on this article (doi:10.1093/eurheartj/ehab516)**

## Introduction

Multimorbidity is associated with reduced quality of life and greater use of health-care resources. It is becoming a global health challenge.^1^ Cardiometabolic multimorbidity (CMM), one of the most replicable multimorbidity profiles,^2^ is defined as the coexistence of two or three cardiometabolic diseases (CMDs), including diabetes, ischaemic heart disease (IHD), and stroke.^3–7^

Although the associations between lifestyle factors (LFs) and single CMDs are well-established, a limited number of studies examined the associations between LFs and CMM. A pooled analysis of 16 cohorts of 120 813 European and US adults showed that the odds ratio [95% confidence interval (CI)] for CMM was 1.9 (1.8–2.3) per 5 kg/m^2^ higher body mass index (BMI) among CMD-free population.^5^ Another study conducted in Nurses’ Health Study and Health Professionals Follow-up Study revealed that the risk of cardiovascular diseases for type 2 diabetes (T2D) patients with three or more low-risk LFs was only half that for patients with 0 low-risk LFs.^8^ Generally, prior studies either investigated the impact of LFs on the development of CMM in participants free of any CMD, regardless of the intermediate progress of a single CMD,^4^^,^^5^^,^^9^ or on the prognosis of patients with a single CMD or CMM.^10^ Although these studies indicated positive effects of LFs, such fragmented analyses by only focusing on one stage of disease progression make it challenging to compare the impact of LFs on different stages before and after a single CMD.

Only one study examined the role of LFs in the progression of CMM in a UK occupational cohort.^4^ Their results showed that LFs had stronger effects on the transition from first CMD (FCMD) to CMM than from CMD-free to FCMD. Whether such results apply to other populations with different genetic and environmental backgrounds, merits further research. Additionally, the majority of studies on LFs and CMM were conducted in the Western population in which the proportion of haemorrhagic stroke (HS) among total stroke is significantly lower than that in the Chinese population.^11^ Since the aetiological pathways and risk factors are not the same for ischaemic stroke (IS) and HS,^12^ the effect estimates of the association of LFs with CMM in the Chinese population may differ.

Therefore, we aimed to examine the associations of single and combined LFs with FCMD, CMM, and death in the China Kadoorie Biobank (CKB) of 0.5 million Chinese adults. Importantly, we used multi-state model to investigate the potentially different impacts of LFs on transitions from free of CMD to FCMD, subsequently to CMM, and further to death. In line with previous studies,^3–7^ we limited CMDs to IHD, stroke (including both IS and HS), and T2D. Because of the large number of cases, we further examined the role of LFs in all possible transitions between healthy and individual CMDs, as well as individual CMDs to CMM and death.

## Methods

### Study design

The CKB is a large-scale prospective cohort of 512 725 participants aged 30–79 years from five urban and five rural areas. All participants provided written informed consent, completed interviewer-administered laptop-based questionnaires, and had physical measurements taken in the 2004–08 baseline survey. Besides long-term outcome follow-up for all participants, periodic resurveys were conducted in 2008 and between 2013 and 2014 in a random sample of about 5% surviving participants (Supplementary material online, *Figure S1*). Information collected from the baseline and resurvey was directly entered into a laptop-based data entry system developed with built-in functions to avoid missing items and to minimize logic errors during the interview. Details of the study design and survey methods have been reported previously.^13^ The study protocol was approved by the Ethics Review Committee of the Chinese Center for Disease Control and Prevention (Beijing, China) and the Oxford Tropical Research Ethics Committee, University of Oxford (UK).

**Figure 1:**
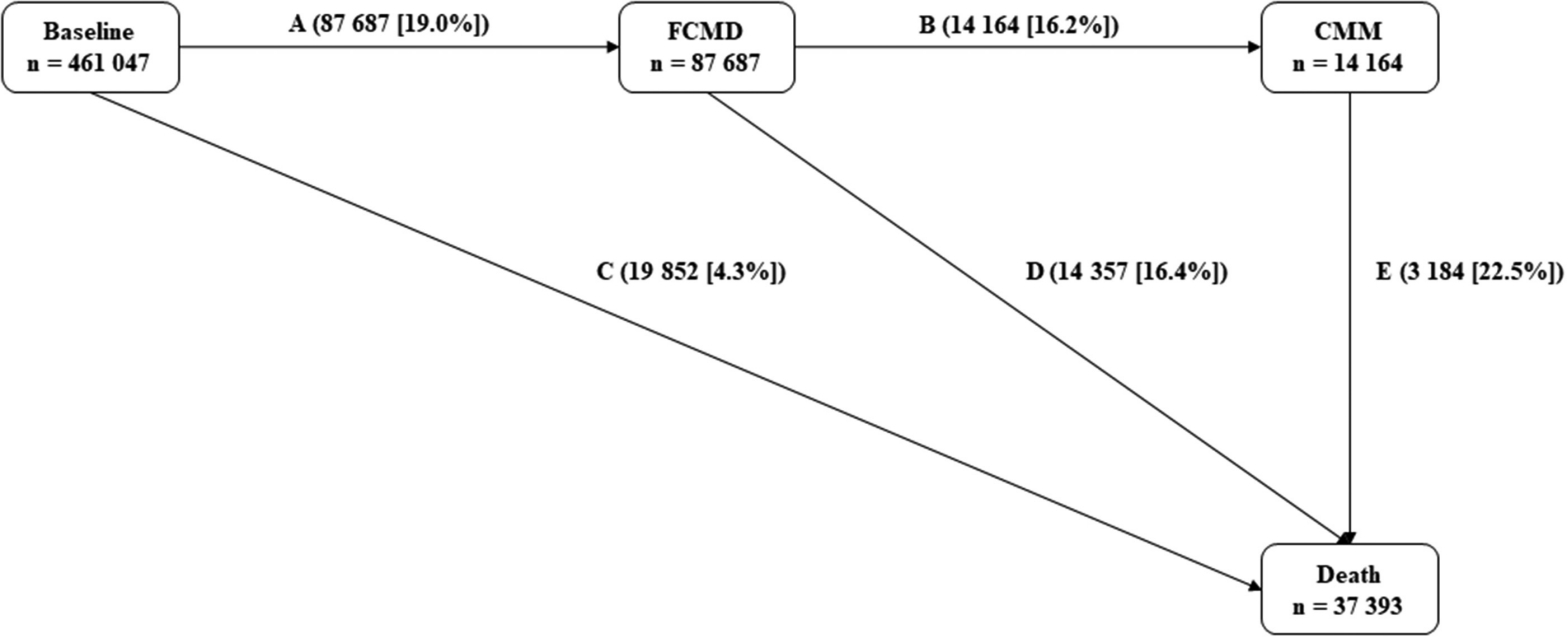
Numbers (percentages) of participants in transition pattern A from baseline to first cardiometabolic disease (FCMD), cardiometabolic multimorbidity (CMM), and death. Cardiometabolic diseases include ischaemic heart disease, stroke, and type 2 diabetes. Cardiometabolic multimorbidity is defined as the occurrence of at least two of the above-mentioned diseases.

We excluded participants who reported history of heart disease (*n* = 15 472), stroke (*n* = 7657), or cancer (*n* = 2385) at baseline. We also excluded those who had self-reported diabetes or screen-detected diabetes, defined as measured fasting blood glucose ≥7.0 mmol/L or random blood glucose ≥11.1 mmol/L at baseline (*n* = 26 162). Participants with missing data for BMI (*n* = 2) were also excluded, leaving 461 047 participants in the present analysis.

### Assessment of lifestyle factors and other covariates

Lifestyle factors of interest were assessed by baseline questionnaires and physical measurements. We asked the frequency, type, and amount of tobacco smoked per day for ever-smokers (former and current). Former smokers were additionally asked about the years since quitting and the reason for quitting. Questions about alcohol consumption included typical drinking frequency, type of alcoholic beverage consumed habitually, and volume of alcohol consumed on a typical drinking day in the past 12 months. For physical activity, the usual type and duration of occupational, commuting, domestic, and leisure time-related activities in the past 12 months were collected. To calculate daily total physical activity level, we multiplied the metabolic equivalent of tasks (METs) for each activity by the hour spent on that activity and summed the MET-hours for all activities.^14^ We assessed habitual intakes of 12 conventional food groups in the past 12 months via a validated qualitative food frequency questionnaire (Supplementary material online, *Table S1*).^15^ Weight, height, and waist circumference (WC) were measured by trained staff using well-calibrated instruments. Body mass index was calculated as weight in kilograms divided by height in metres squared.

**Table 1:** Hazard ratios (95% CIs) for each transition in transition pattern B by lifestyle factors among 459 606 participants

	HRs (95% CIs)					
	No. of events	Tobacco smoking	Excessive alcohol drinking	Less healthy dietary habits	Low physical activity	Unhealthy body shape
Baseline → FCMD						
Baseline → IHD	34 304	**1.23 (1.19–1.27)**	1.03 (0.99–1.07)	1.06 (0.99–1.13)	**1.10 (1.07–1.12)**	**1.33 (1.30–1.36)**
Baseline → IS	31 012	**1.14 (1.10–1.18)**	**1.21 (1.17–1.26)**	**1.23 (1.14–1.33)**	**1.08 (1.06–1.11)**	**1.19 (1.17–1.22)**
Baseline → HS	6715	0.99 (0.92–1.05)	**1.30 (1.20–1.40)**	1.20 (0.95–1.52)	**1.16 (1.10–1.22)**	**1.16 (1.10–1.23)**
Baseline → T2D	14 215	0.98 (0.93–1.04)	0.99 (0.94–1.05)	1.10 (0.96–1.26)	**1.08 (1.04–1.12)**	**2.47 (2.39–2.56)**
FCMD→ CMM						
IHD → CMM	5507	1.04 (0.97–1.13)	**1.18 (1.08–1.29)**	1.00 (0.86–1.16)	**1.10 (1.04–1.17)**	**1.27 (1.20–1.34)**
IS → CMM	5968	1.06 (0.99–1.14)	0.96 (0.88–1.05)	**1.25 (1.05–1.49)**	1.05 (1.00–1.11)	**1.23 (1.16–1.29)**
HS → CMM	1088	**0.82 (0.68–0.99)**	1.00 (0.82–1.23)	**1.84 (1.04–3.25)**	**1.22 (1.06–1.40)**	1.08 (0.95–1.24)
T2D → CMM	2094	**1.21 (1.07–1.38)**	**1.21 (1.05–1.39)**	0.95 (0.69–1.31)	1.05 (0.95–1.15)	**1.11 (1.02–1.22)**
Baseline → Death	19 852	**1.40 (1.35–1.45)**	**1.27 (1.22–1.32)**	1.02 (0.91–1.14)	**1.17 (1.13–1.21)**	**1.11 (1.08–1.15)**
FCMD → Death						
IHD → Death	5819	**1.26 (1.18–1.36)**	1.06 (0.98–1.15)	1.11 (0.91–1.35)	**1.23 (1.16–1.31)**	1.05 (0.99–1.11)
IS → Death	3107	**1.28 (1.17–1.41)**	1.09 (0.98–1.20)	1.12 (0.83–1.50)	**1.26 (1.16–1.36)**	1.03 (0.95–1.11)
HS → Death	3671	1.05 (0.95–1.16)	0.95 (0.86–1.06)	1.42 (0.97–2.07)	**1.24 (1.15–1.34)**	**1.10 (1.02–1.19)**
T2D → Death	776	**1.38 (1.13–1.68)**	**1.45 (1.19–1.76)**	1.12 (0.54–2.30)	**1.20 (1.03–1.41)**	0.94 (0.81–1.09)
CMM → Death	3810	**1.19 (1.10–1.30)**	**1.14 (1.04–1.26)**	1.11 (0.86–1.43)	1.03 (0.96–1.10)	1.00 (0.93–1.07)

Cardiometabolic diseases include IHD, IS, HS, and T2D. Cardiometabolic multimorbidity is defined as the occurrence of at least two of the above-mentioned diseases.

Number of events refers to number of cases in each transition.

Multivariable models were stratified by age in the 5-year interval, study area, and adjusted for sex, education, marital status, parental family history of CMM. For analyses of dichotomous lifestyle factors, five lifestyle factors were mutually adjusted. Values shown in bold are statistically significant (*P* < 0.05).

High-risk lifestyle factors were defined as follows: current smoking or having stopped because of illness; daily drinking ≥30 g/day of pure alcohol or having stopped drinking habit; non-daily eating of vegetables, fruits, and eggs, and eating red meat daily or less than weekly; engaging in a sex- and age-specific lower half of total physical activity; having BMI <18.5 or ≥28.0 kg/m^2^ or having waist circumference ≥90 cm (men)/85 cm (women).

CI, confidence interval; CMM, cardiometabolic multimorbidity; HR, hazard ratio; HS, haemorrhagic stroke; IHD, ischaemic heart disease; IS, ischaemic stroke; T2D, type 2 diabetes.

A range of covariates were also assessed by the baseline questionnaire, including socio-demographic characteristics, personal and family medical history, and female reproductive information. Participants who had at least one parent suffering from chronic heart disease, stroke, or diabetes were considered as having a CMD family history. Of those, participants who reported at least one parent suffering from at least two of the CMDs were considered as having a CMM family history. Prevalent hypertension was defined as measured systolic blood pressure ≥140 mmHg, measured diastolic blood pressure ≥90 mmHg, a self-reported diagnosis of hypertension, or antihypertensive medication use at baseline.

### Definition of high-risk lifestyle factors

We considered five LFs, including smoking, alcohol drinking, dietary habit, physical activity, and body shape according to previous studies.^16^^,^^17^ The definitions for high-risk LFs were according to the data availability of our study and the characteristics of the Chinese population. Their associations with cardiovascular disease, diabetes, and mortality are well-established in our population.^15^^,^^18^^,^^19^ Also, we did not consider clinical factors such as total cholesterol, blood pressure, and glucose, as included in the well-known Life’s Simple 7,^20^ because they are potential mediators between LFs and CMDs.

For smoking, we assigned current smokers and former smokers who quit because of illness to the high-risk group. For alcohol drinking, the high-risk group was defined as those who drank ≥30 g/day of pure alcohol or having stopped drinking. Former smokers and drinkers were included in the high-risk group to avoid a misleadingly elevated risk for the reference group. For physical activity, we defined the high-risk group as those who engaged in a sex- and age- (<50, 50–59, and ≥60 years) specific lower half of total physical activity. According to the Chinese dietary guidelines^21^ and previous findings in our population,^22^ we defined unhealthy dietary habits as non-daily eating of vegetables, fruits, and eggs, and eating red meat daily or less than weekly. For body shape, both BMI and WC were considered to reflect energy balance.^20^ Participants having BMI <18.5 or ≥28.0 kg/m^2^ or having WC ≥90 cm (men)/85 cm (women) were considered as high risk, which emphasized avoidance of extremely high or low weight and abdominal obesity. The number of high-risk LFs was counted, ranging from 0 to 5.

### Follow-up for cardiometabolic disease and death

Incident cases of interest and death were identified through linkages to disease and mortality registries and national health insurance claim database, supplemented with local residential records and annual active confirmation. Causes of death were ascertained chiefly by death certificates and supplemented by reviews of medical records and verbal autopsy using validated instruments. All events were coded according to the International Classification of Diseases, 10th Revision by trained staff blinded to baseline information.

In the present analysis, IHD, IS, and HS were defined by code I20 to I25, I63, and I61. For T2D, after excluding cases with a definitive diagnosis of insulin-dependent, malnutrition-related, or other specified diabetes (E10, E12, and E13), we included cases coded as E11 or E14 in the analysis. Because participants in our study were aged mostly over 40 years at baseline, it was reasonable to assume that incident cases of unspecified diabetes (E14) were mostly T2D. Since 2014, medical records of incident IHD and stroke cases were retrieved and reviewed by qualified cardiovascular specialists blinded to baseline exposures of patients. By October 2018, of 33 515 retrieved medical records of IHD cases and 40 465 retrieved medical records of stroke cases, the diagnosis was confirmed in 87.9% of IHD cases and 91.8% of stroke cases. The medical records of a random sample of 831 diabetes cases were retrieved and reviewed, and 98.6% were confirmed for diagnosis. Therefore, we did not conduct further adjudication for diabetes.

### Statistical methods

Participants were considered at risk from enrolment to death, loss to follow-up, or 31 December 2017, whichever came first. Changes in dichotomized LFs by the number of CMDs that occurred between 2004–08 baseline and 2013–14 resurvey were analysed with adjustment for age, sex, and study area, as appropriate (Supplementary material online, *Methods*).

In the following analyses, age was used as the time scale. The models were stratified by age in 5-year interval and study area, and adjusted for sex, education, marital status, menopausal status (women only), and parental family history of CMM. We first used Cox proportional hazards model to estimate the hazard ratios (HRs) and 95% CIs of high-risk LFs (individual or combined) with FCMD, CMM, and all-cause death. In the analyses of individual LFs, the model included five LFs simultaneously. The number of high-risk LFs entered the model as a categorical variable, with 0–1 high-risk LFs being the reference group; this variable also entered a separate model as an ordinal variable to assess the HR of events of interest per one-factor increase in the number of LFs.

We further used multi-state model to assess the role of both individual and combined LFs in the temporal disease progression from free of CMD to FCMD, CMM, and death.^23^ The multi-state model is an extension of the competing risk model and it is useful to explore how certain factors influence different phases of a process.^24^ Five transition stages were constructed based on the natural history of CMM and a previous study^4^ (transition pattern A, *Figure 1*): (i) baseline healthy to FCMD; (ii) FCMD to CMM; (iii) baseline healthy to death from a disease other than IHD, stroke, and T2D; (iv) FCMD to death from any causes; and (v) CMM to death from any causes. For participants who entered different states on the same date (*n* = 8365), we calculated the entering date of the theoretically prior state as the entering date of the latter state minus 0.5 day. For example, for participants who died of FCMD, the date of FCMD occurrence equals the date of death minus 0.5 day.

**Figure 2:**
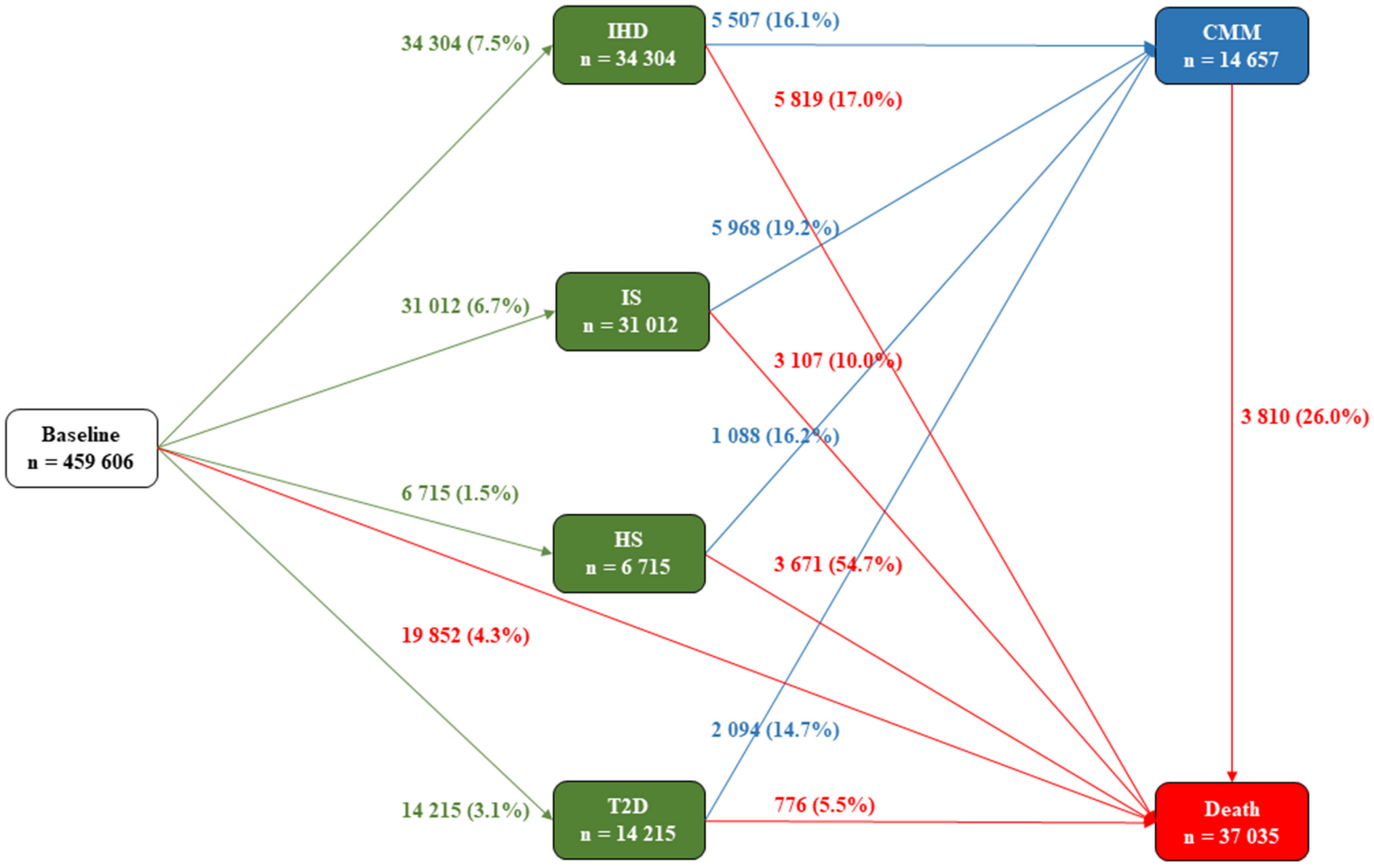
Numbers (percentages) of participants in transition pattern B from baseline to one of IHD, IS, HS, and T2D, then to cardiometabolic multimorbidity (CMM), and subsequently to death. Cardiometabolic diseases include ischaemic heart disease (IHD), ischaemic stroke (IS), haemorrhagic stroke (HS), and type 2 diabetes (T2D). Cardiometabolic multimorbidity is defined as the occurrence of at least two of the above-mentioned diseases.

We also used the multi-state model with the same setting to analyse the effects of LFs on different pathways from baseline to CMM, and to the absorbing state—death. We divided the FCMDs into four individual diseases, i.e. IHD, IS, HS, and T2D, resulting in 14 transitions (transition pattern B, *Figure 2*). In this pattern, CMM was redefined as having two of four individual diseases. Because we could not ascertain the temporal sequence of disease occurrences if a participant was diagnosed with at least two of IHD, IS, HS, and T2D on the same date, we excluded them, leaving 459 606 participants in this analysis.

We predicted the probabilities of becoming an FCMD survivor, CMM survivor, dead without CMD, dead with FCMD, dead with CMM from the enrolment to the longest follow-up. Probabilities were separately obtained for participants who had 0–1 and five high-risk LFs, with all covariates being set to the average level of the CKB population in the present analysis.

Several sensitivity analyses for the multi-state analyses of the transition pattern A were conducted: (i) calculating the entering date of the prior state using different time intervals (0.5, 1, 3, and 5 years) for participants who entered different states on the same day; (ii) excluding participants who entered different states on the same date; (iii) additionally adjusting for hypertension, usage of blood pressure medicine, and statin at baseline; (iv) including participants who had previously diagnosed heart disease, stroke, or diabetes, and assigning them to FCMD or CMM state according to their CMD status at baseline; (v) excluding the events occurring in the first 2 years of follow-up; and (vi) in addition to the predefined five transitions, adding another transition from the baseline directly to CMM.

The multistate analyses of the transition pattern A were further stratified according to sex, age, residence area, family history of CMD, and hypertension. The interactions were tested by using the likelihood ratio test comparing models with and without a cross-product term.

The multi-state model was performed using ‘mstate’ package of R (version 3.6.3), and all other analyses were performed using Stata (version 14, StataCorp). Two-tailed *P *<* *0.05 indicated statistical significance.

## Results

### Descriptive analysis

The mean age of 461 047 participants was 51.2 ± 10.5 years. Of those, 41.0% were male, and 42.3% resided in urban areas. During a median follow-up of 11.2 years [interquartile range 10.2–12.1 years; total person-years (PYs) 5 032 900], 87 687 participants experienced at least one CMD, with a crude incidence rate of 187.59 per 10 000 PYs (*Figure 1*). Of all incident CMD patients, 14 164 (transition B, 445.88/10 000PYs) developed CMM, and afterward, 3184 (transition E, 780.44/10 000 PYs) died from any causes; 19 852 (transition C, 42.47/10 000PYs) died without experiencing CMM. The primary cause of death in transition C was cancer (ICD-10: C00-C97), accounting for 54.1% of deaths. Disease of the circulatory system (ICD-10: I00-I99) was the primary cause of death in both transitions D and E, accounting for more than 70% of deaths.

In the separate analysis for individual diseases (transition pattern B; 1441 participants were excluded), 34 304 (7.5% of the baseline healthy participants) participants’ FCMDs were IHD, 31 012 (6.7%) were IS, 6715 (1.5%) were HS, and 14 215 (3.1%) were T2D (*Figure 2*). Compared with cases whose FCMD was IHD, IS, or T2D, cases whose FCMD was HS were more likely to die afterwards, with 3671 (54.7%) died during follow-up.

Overall, men were more likely than women to have high-risk lifestyles, with 55.4% of men and 17.1% of women having three or more high-risk LFs. Cardiometabolic multimorbidity survivors and participants who died with CMM were more likely to be older, urban resident, and to have hypertension and more high-risk LFs than others (Supplementary material online, *Table S2* and *S3*). Among 22 965 participants who participated in both the 2004–08 baseline and 2013–14 resurvey, most had not changed their risk level of lifestyles. Participants between different CMD status groups exhibited no distinct differences in the changes in lifestyles (Supplementary material online, *Table S4*).

**Table 2:** Hazard ratios (95% CIs) for each transition in transition pattern B by number of high-risk lifestyle factors among 459 606 participants

	No. of events	HRs (95% CIs)					
		0–1	2	3	4	5	Ordinal scale
Baseline → FCMD							
Baseline → IHD	34 304	Reference	**1.15 (1.12–1.19)**	**1.38 (1.33–1.43)**	**1.63 (1.55–1.71)**	**2.02 (1.85–2.20)**	**1.18 (1.17–1.20)**
Baseline → IS	31 012	Reference	**1.11 (1.08–1.15)**	**1.28 (1.23–1.32)**	**1.53 (1.46–1.61)**	**1.85 (1.69–2.03)**	**1.15 (1.14–1.17)**
Baseline → HS	6715	Reference	1.05 (0.98–1.13)	**1.24 (1.15–1.34)**	**1.50 (1.36–1.66)**	**1.69 (1.38–2.06)**	**1.15 (1.12–1.18)**
Baseline → T2D	14 215	Reference	**1.40 (1.34–1.47)**	**2.03 (1.93–2.14)**	**2.60 (2.42–2.81)**	**4.26 (3.76–4.82)**	**1.40 (1.38–1.43)**
FCMD→ CMM							
IHD → CMM	5507	Reference	**1.11 (1.02–1.20)**	**1.29 (1.19–1.40)**	**1.57 (1.40–1.76)**	**1.60 (1.32–1.95)**	**1.15 (1.11–1.18)**
IS → CMM	5968	Reference	**1.19 (1.10–1.29)**	**1.29 (1.18–1.40)**	**1.33 (1.18–1.48)**	**1.53 (1.26–1.85)**	**1.10 (1.07–1.13)**
HS → CMM	1088	Reference	1.05 (0.88–1.26)	1.12 (0.92–1.36)	1.12 (0.85–1.47)	1.60 (0.98–2.60)	1.07 (0.99–1.15)
T2D → CMM	2094	Reference	**1.14 (1.00–1.30)**	**1.25 (1.09–1.44)**	**1.34 (1.11–1.62)**	**1.79 (1.33–2.43)**	**1.12 (1.06–1.18)**
Baseline → Death	19 852	Reference	**1.17 (1.12–1.22)**	**1.39 (1.33–1.45)**	**1.81 (1.71–1.92)**	**2.13 (1.92–2.36)**	**1.21 (1.19–1.23)**
FCMD → Death							
IHD → Death	5819	Reference	**1.16 (1.07–1.27)**	**1.33 (1.22–1.45)**	**1.62 (1.45–1.81)**	**1.46 (1.21–1.75)**	**1.14 (1.11–1.18)**
IS → Death	3107	Reference	**1.20 (1.06–1.35)**	**1.34 (1.19–1.51)**	**1.57 (1.35–1.83)**	**1.86 (1.45–2.39)**	**1.16 (1.11–1.20)**
HS → Death	3671	Reference	**1.14 (1.03–1.26)**	**1.24 (1.10–1.38)**	**1.30 (1.12–1.51)**	**2.04 (1.53–2.71)**	**1.11 (1.07–1.16)**
T2D → Death	776	Reference	1.08 (0.86–1.34)	1.21 (0.96–1.53)	**1.52 (1.14–2.02)**	**2.58 (1.69–3.93)**	**1.19 (1.10–1.29)**
CMM → Death	3810	Reference	0.96 (0.86–1.06)	1.10 (0.99–1.22)	**1.25 (1.10–1.44)**	1.00 (0.79–1.27)	**1.07 (1.03–1.11)**

Cardiometabolic diseases include IHD, IS, HS, and T2D. Cardiometabolic multimorbidity is defined as the occurrence of at least two of the above-mentioned diseases.

Number of events refers to number of cases in each transition.

Multivariable models were stratified by age in the 5-year interval, study area, and adjusted for sex, education, marital status, parental family history of CMM. When the number of lifestyle factors was included as a categorical variable, the reference group was those having 0–1 lifestyle factors. Values shown in bold are statistically significant (*P* < 0.05).

High-risk lifestyle factors were defined as follows: current smoking or having stopped because of illness; daily drinking ≥30 g/day of pure alcohol or having stopped drinking habit; non-daily eating of vegetables, fruits, and eggs, and eating red meat daily or less than weekly; engaging in a sex- and age-specific lower half of total physical activity; having BMI <18.5 or ≥28.0 kg/m^2^ or having waist circumference ≥90 cm (men)/85 cm (women).

CI, confidence interval; CMM, cardiometabolic multimorbidity; HR, hazard ratio; HS, haemorrhagic stroke; IHD, ischaemic heart disease; IS, ischaemic stroke; T2D, type 2 diabetes.

### Cox regression analyses

All five high-risk LFs were associated with an increased risk of FCMD, CMM, and all-cause death (Supplementary material online, *Table S5*). Body shape defined by BMI and WC showed the strongest associations with both FCMD and CMM [HRs (95% CIs): 1.40 (1.38, 1.42) and 1.63 (1.57, 1.68)]. Smoking showed the strongest association with death, with an HR (95% CI) of 1.35 (1.31, 1.38). Upward trends were observed for risks of FCMD, CMD, and death with an increasing number of high-risk LFs; the corresponding HRs (95% CIs) per one-factor increase was 1.20 (1.19, 1.21), 1.29 (1.27, 1.32), and 1.23 (1.21, 1.24), respectively. Generally, the effect estimates for associations were slightly stronger for transition to CMM than that for transition to FCMD.

### Multi-state analyses

**Figure 3:**
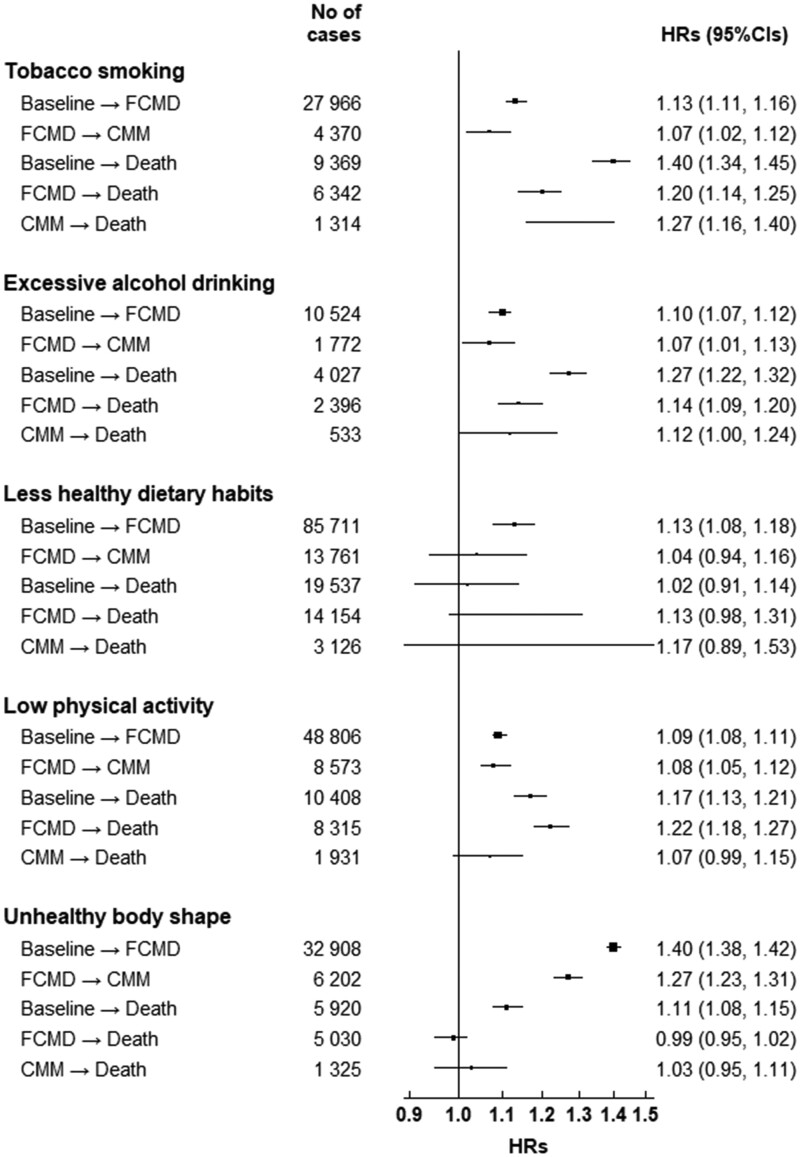
Hazard ratios (95% CIs) for transition pattern A by lifestyle factors among 461 047 participants. CI, confidence interval; CMM, cardiometabolic multimorbidity; FCMD, first cardiometabolic disease; HR, hazard ratio. Cardiometabolic diseases include ischaemic heart disease, stroke, and type 2 diabetes. Cardiometabolic multimorbidity is defined as the occurrence of at least two of the above-mentioned diseases. Number of cases refers to number of cases in each transition with the corresponding exposure. Multivariable models were stratified by age in the 5-year interval, study area, and adjusted for sex, education, marital status, and parental family history of cardiometabolic multimorbidity. For analyses of dichotomous lifestyle factors, five lifestyle factors were mutually adjusted. High-risk lifestyle factors were defined as follows: current smoking or having stopped because of illness; daily drinking ≥30 g/day of pure alcohol or having stopped drinking habit; non-daily eating of vegetables, fruits, and eggs, and eating red meat daily or less than weekly; engaging in a sex- and age-specific lower half of total physical activity; having BMI <18.5 or ≥28.0 kg/m^2^ or having waist circumference ≥90 cm (men)/85 cm (women).

Multi-state analyses showed the same result for the transition from baseline (free of CMD) to FCMD as the Cox regression analyses, but further distinguished the roles of high-risk LFs in the temporal trajectories of CMM (*Figure 3*). The impacts of smoking, unhealthy dietary habits, and unhealthy body shape on the transition to FCMD were stronger than those on the transition to subsequent CMM. The effect estimates of four LFs, except for physical inactivity, were similar for the transition from FCMD to death and from CMM to death. Besides, the strength of associations of smoking, excessive drinking, and physical inactivity with mortality outcome (either from healthy, FCMD, or CMM) was greater than that of morbidity outcome. In contrast, the opposite was observed for unhealthy body shape.

**Figure 4:**
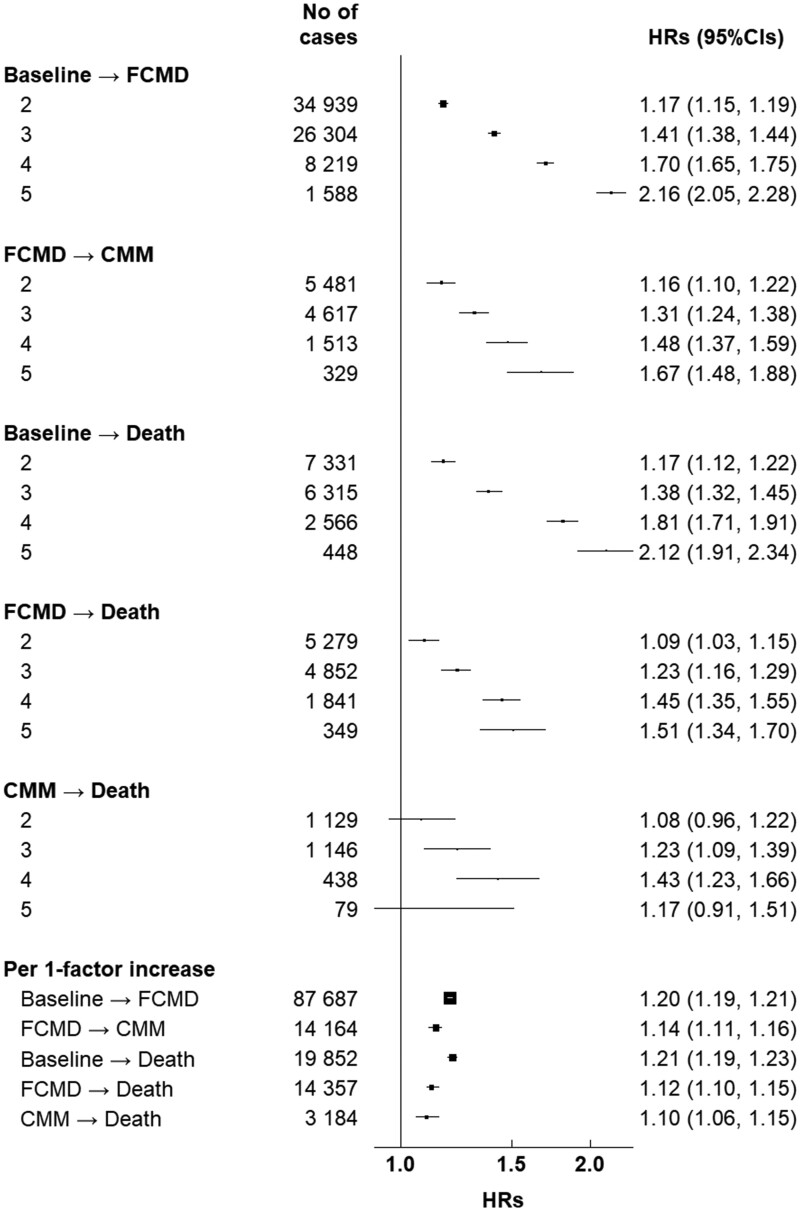
Hazard ratios (95% CIs) for transition pattern A by number of high-risk lifestyle factors among 461 047 participants. CI, confidence interval; CMM, cardiometabolic multimorbidity; FCMD, first cardiometabolic disease; HR, hazard ratio. Cardiometabolic diseases include ischaemic heart disease, stroke, and type 2 diabetes. Cardiometabolic multimorbidity is defined as the occurrence of at least two of the above-mentioned diseases. Number of cases refers to number of cases in each transition with the corresponding exposure. Multivariable models were stratified by age in the 5-year interval, study area, and adjusted for sex, education, marital status, parental family history of cardiometabolic multimorbidity. When the number of lifestyle factors was included as a categorical variable, the reference group was those having 0–1 lifestyle factors. High-risk lifestyle factors were defined as follows: current smoking or having stopped because of illness; daily drinking ≥30 g/day of pure alcohol or having stopped drinking habit; non-daily eating of vegetables, fruits, and eggs, and eating red meat daily or less than weekly; engaging in a sex- and age-specific lower half of total physical activity; having BMI <18.5 or ≥28.0 kg/m^2^ or having waist circumference ≥90 cm (men)/85 cm (women).

When LFs were combined, gradients in the associations were observed between the number of high-risk LFs and all five transitions (*Figure 4*). Also, we observed a stronger association of the number of LFs with the transition from healthy to FCMD vs. the subsequent transition to CMM, and a similar magnitude of the associations with mortality outcome (either from FCMD or CMM). The adjusted HRs (95% CIs) per one-factor increase were 1.20 (1.19, 1.21), 1.14 (1.11, 1.16), 1.21 (1.19, 1.23), 1.12 (1.10, 1.15), and 1.10 (1.06, 1.15) for transitions A–E, respectively.

After dividing FCMDs into four specific CMDs (i.e. IHD, IS, HS, and T2D), we found that physical inactivity was significantly associated with basically all possible transitions (*Table 1*). Smoking was more likely to be associated with mortality risk and incidence of IHD and IS than with HS and T2D. Unhealthy body shape had fewer impacts on transitions from FCMD or CMM to death than other transitions. We also observed gradients in associations of combined LFs with all transitions except for the transition from HS to CMM (*Table 2*).

At the longest follow-up of 13.5 years, given the average level of covariates in the CKB population, 16.2%, 1.8%, and 1.7% of participants with 0–1 high-risk LFs at baseline were predicted to develop at least one CMD (after that, survived or died), CMM (after that, survived or died), and die with CMD (including FCMD and CMM) (Supplementary material online, *Figure S2*). In contrast, having five high-risk LFs approximately doubled the risk of developing CMD (31.9%), tripled the risk of CMM (5.8%), and tripled the risk of death with CMD (5.0%).

### Sensitivity and stratified analyses

The results were not substantially altered in the sensitivity analyses (Supplementary material online, *Tables S6 and S7*). Although several statistically significant interactions were found in the stratified analyses, most of them seemed clinically meaningless (Supplementary material online, *Figures S3 and S4*). When the analysis was stratified by residence area or family history of CMD, statistically significant modification effects were only found on one of the five transitions. Prevalent hypertension modified the associations of the number of high-risk LFs with three stages of transition, but with a different direction. In contrast, we observed similar impacts of age on the transitions between high-risk LFs and morbidity transitions. Taking those with 0–1 high-risk LFs as the reference, the associations of having 4–5 factors with incidence transitions were stronger among younger participants.

## Discussion

The present prospective study of 0.5 million Chinese adults found that five high-risk LFs played important roles in all disease transition stages from healthy to FCMD, to CMM, and then to death, but to different extents. The associations of LFs with the transition from healthy to FCMD were stronger than those with the transition from FCMD to CMM. Also, a stronger impact of LFs on morbidity outcomes (i.e. healthy to FCMD and FCMD to CMM) was observed among middle-aged than older participants. When we further divided FCMDs into four individual CMDs, we found that single LFs had different impacts on disease-specific transitions even within the same transition stage (e.g. four possible disease-specific transitions from baseline healthy to IHD, IS, HS, and T2D) (*Graphical abstract*).

Three prior studies conducted in European, US, and Australian populations estimated the associations of LFs with FCMD and CMM among participants free of CMD at baseline.^4^^,^^5^^,^^9^ Findings from a prospective cohort of 13 174 Australian women aged 45–50 revealed hazardous impacts of being overweight or obese, physically inactive, and current smokers on developing FCMD and even stronger effect estimates for developing CMM.^9^ A study conducted in the Whitehall II cohort of 8270 middle-aged UK participants found that current smoking, alcohol abstention or heavy alcohol consumption, low consumption of fruit and vegetable, and BMI ≥25.0 kg/m^2^ were associated with both FCMD and CMM. Compared with participants without any high-risk LFs, the HRs (95% CIs) for those with four high-risk LFs (except for BMI) were 1.64 (1.26–2.13) and 3.09 (1.83–5.22) for FCMD and CMM, respectively.^4^ A pooled analysis of individual data from 16 European and US cohorts showed that higher BMI was associated with a higher risk of CMM, with an odds ratio (95% CI) of 1.9 (1.8–2.3) per 5 kg/m^2^ increment.^5^

Using the same analysis strategy as the above studies, we also observed a slightly stronger association of LFs with CMM than FCMD. Although this analysis strategy is conventional for studies on risk factors for multimorbidity,^1^ some limitations merit consideration. First, this strategy uses participants free of CMD at baseline to directly analyse the association between LFs and the subsequent risk of CMM. Those who develop only one CMD and survive or die thereafter during the follow-up period were simply regarded as censored. We find it hard to distinguish whether the LFs have a different impact on the transition from healthy to FCMD, from FCMD to CMM, and from FCMD to death. Second, given the importance of LFs for mortality risk, participants who died from any causes, including their FCMD, had a greater risk of developing CMM than those who remained alive. The competing risk from death leads to a violation of the independent censoring assumption and could alter the risk estimates.^23^

Hence, we used the multi-state model, an extension of the competing risk model, to distinguish the difference in impacts of LFs on the five transitions between healthy, FCMD, CMM, and death. We observed that LFs had influences on each transition, but to different extents. The only study using a similar analytic method was based on the Whitehall II cohort in which the authors found that LFs had a relatively greater role in the transition from FCMD to CMM than from healthy to FCMD.^4^ Compared with having no high-risk LFs, the HRs (95% CIs) of having four high-risk factors were 2.00 (1.40–2.85) and 1.44 (1.22–1.69) for the transition from FCMD to CMM and transition from healthy to FCMD, respectively. In contrast, we found that high-risk LFs conferred a relatively greater risk for transition to FCMD than to CMM.

Besides LFs, several other factors affect the progression from FCMD to CMM. They may cover up the relative importance of LFs in determining the risk of developing CMM, to some extent. A systematic analysis from the Global Burden of Disease 2016 concluded that health-care access and quality in China was relatively lower than that in UK.^25^ In China, only 32.2% of diabetes patients were treated, and 15.8% were controlled^26^; 30.1% of hypertensive patients were treated, and 7.2% were controlled.^27^ Even among patients with IHD or stroke, only half took secondary prevention medicine.^28^ Nonetheless, LFs were still non-negligible contributors to the occurrence of CMM. Our results confirm the well-known role of LFs in the primary prevention for CMDs^29^^,^^30^ and further provide evidence that there is potential for lifestyle intervention in contributing to the secondary prevention of CMDs. In the era of ageing and multimorbidity, it is of great significance to reduce the rising medication cost and burden of polypharmacy.

Our previous studies in the CKB population estimated that 43.2% IHD, 39.1% IS, and 78.8% T2D were preventable by adherence to a healthy lifestyle.^15^^,^^18^^,^^19^ In this study, we have extended these findings from a single disease to multimorbidity. The probabilities of occurring CMD and CMM for participants having five high-risk LFs were about two times and three times as high as those having 0–1 LF, suggesting an enormous preventive potential of lifestyle intervention in the whole progression of the disease. Our stratified analysis further revealed that LFs had a relatively larger effect on the risks of FCMD and subsequent CMM among middle-aged than older participants; and both effect sizes were similar among middle-aged participants. It is probably due to the relatively higher baseline hazard for older participants and the survival effect, i.e. participants at high risk for death had already died before the enrolment. These findings indicate greater significance in strengthening lifestyle interventions among younger adults.

Limited by sample size, no previous study was able to further detail the progression of four specific CMDs and examined the role of LFs in all possible transitions between healthy and individual CMDs, as well as individual CMDs to CMM and death as our study. We found that single LFs had distinct impacts on different transition stages. For example, an unhealthy body shape was associated with increased risks of almost all morbidity outcomes (i.e. baseline to FCMD and FCMD to CMM) but was not associated with the risk of death among CMD patients. And even within a certain transition stage, single LFs had different impacts on disease-specific transitions. For example, smoking was more specifically related to the risk of ischaemic cardiovascular disease (i.e. IHD and IS) than to HS and T2D. In contrast, our study observed that total physical activity (including occupational, commuting, domestic, and leisure time physical activity) played roles in basically all transitions of progression of CMDs, suggesting a universal benefit of being physically active.

The present study provides evidence from a uniquely only Chinese cohort living in China, an underrepresented population under a health-care system different from that of developed countries where most previous studies were conducted. The chief strength of our study lies in the use of the multi-state model, yielding less biased estimates than the conventional Cox model and distinguishing the impact of LFs on each transition in the progression trajectory of CMD. It is also one of the largest prospective studies on associations of LFs with CMM worldwide. The large number of cases recorded during more than 10 years of follow-up allowed us to model all possible transitions from baseline healthy to one of the four FCMDs, and then to CMM and death. Also, the study includes a nationwide geographically spread population, with a broad range of ages and diverse socio-demographic characteristics. By performing thorough investigation of LFs and covariates and extensive sensitivity analyses, the study’s internal validity was also enhanced.

Some limitations characterize the present study. First, we used LFs collected at baseline and did not account for possible changes during follow-up. However, we conducted a separate analysis in participants attending both the baseline survey and 2013–14 resurvey. During a median interval of around 8 years, most of them had not changed their risk level of lifestyles. The change in LFs was similar between those who developed CMD or CMM during the follow-up and those who did not. The use of baseline LFs may also help avoid reverse causation resulted from lifestyle change after disease onset. Second, we did not collect information on the treatment for CMD, of which medication adherence and compliance might correlate to the adherence to a healthy lifestyle. However, education, as a surrogate for several health-related behaviours, has been adjusted. The residual confounding from treatment is less likely to completely explain the relative risk of LFs with the transition from FCMD to CMM and subsequent death observed in the current study.^31^ Third, some participants were newly diagnosed with two CMDs simultaneously in one admission, causing the same diagnosis date for both CMDs. We used an interval of 0.5 day to calculate the onset date of FCMD, which might lead to an inaccurate association. However, we tried several different intervals, dropped the same-day participants, and added a 6th transition directly from baseline to CMM. None of these additional analyses altered the results significantly. Fourth, the observational nature of our study precluded causal inference. However, the casual associations of LFs with single CMDs are supported by emerging evidence from prospective cohort studies,^10^^,^^32^ Mendelian randomization studies,^33^^,^^34^ and randomized controlled trials.^35^^,^^36^ Nevertheless, the casual roles of modifiable LFs in the prognosis of CMDs need further research. Other limitations included information bias caused by self-reported LFs and residual confounding due to unmeasured or unknown factors.

In conclusion, this large-scale prospective cohort of Chinese adults revealed that LFs differently influenced the progression from healthy to FCMD, to CMM, and further to death, and also had diverse impacts on disease-specific transitions. Our findings further add to the evidence that it is of great significance to integrate comprehensive lifestyle interventions into both health management and cardiometabolic disease management programmes if observed associations are causal. Further studies are warranted on whether genetic susceptibility modifies the effects of LFs on the development and prognosis of CMM and which cardiometabolic biomarkers may mediate the effect of LFs. 

## Supplementary material

Supplementary material is available at *European Heart Journal* online.

## Supplementary Material

ehab413_Supplementary_Materials

## Data Availability

Details of how to access China Kadoorie Biobank data and details of the data release schedule are available from www.ckbiobank.org/site/Data+Access.
