# Supplementary material for: Lifestyle, cardiometabolic disease, and multimorbidity in a prospective Chinese study
Source: Eur Heart J. 2021 Aug 1;42(34):3374–84. doi: 10.1093/eurheartj/ehab413 (PMC8423468; doi:10.1093/eurheartj/ehab413)
Supplement: ehab413_Supplementary_Materials [file ehab413_supplementary_materials.docx]

# Supplementary material

Members of the China Kadoorie Biobank collaborative group:

**International Steering Committee:** Junshi Chen, Zhengming Chen (PI), Robert Clarke, Rory Collins, Yu Guo, Liming Li (PI), Jun Lv, Richard Peto, Robin Walters. **International Co-ordinating Centre, Oxford:** Daniel Avery, Ruth Boxall, Derrick Bennett, Yumei Chang, Yiping Chen, Zhengming Chen, Robert Clarke, Huaidong Du, Simon Gilbert, Alex Hacker, Mike Hill, Michael Holmes, Andri Iona, Christiana Kartsonaki, Rene Kerosi, Ling Kong, Om Kurmi, Garry Lancaster, Sarah Lewington, Kuang Lin, John McDonnell, Iona Millwood, Qunhua Nie, Jayakrishnan Radhakrishnan, Paul Ryder, Sam Sansome, Dan Schmidt, Paul Sherliker, Rajani Sohoni, Becky Stevens, Iain Turnbull, Robin Walters, Jenny Wang, Lin Wang, Neil Wright, Ling Yang, Xiaoming Yang. **National Co-ordinating Centre, Beijing:** Zheng Bian, Yu Guo, Xiao Han, Can Hou, Jun Lv, Pei Pei, Chao Liu, Canqing Yu. **10 Regional Co-ordinating Centres: Qingdao CDC:** Zengchang Pang, Ruqin Gao, Shanpeng Li, Shaojie Wang, Yongmei Liu, Ranran Du, Yajing Zang, Liang Cheng, Xiaocao Tian, Hua Zhang, Yaoming Zhai, Feng Ning, Xiaohui Sun, Feifei Li. **Licang CDC:** Silu Lv, Junzheng Wang, Wei Hou. **Heilongjiang Provincial CDC:** Mingyuan Zeng, Ge Jiang, Xue Zhou. **Nangang CDC:** Liqiu Yang, Hui He, Bo Yu, Yanjie Li, Qinai Xu,Quan Kang, Ziyan Guo. **Hainan Provincial CDC:** Dan Wang, Ximin Hu, Jinyan Chen, Yan Fu, Zhenwang Fu, Xiaohuan Wang. **Meilan CDC:** Min Weng, Zhendong Guo, Shukuan Wu,Yilei Li, Huimei Li, Zhifang Fu. **Jiangsu Provincial CDC:** Ming Wu, Yonglin Zhou, Jinyi Zhou, Ran Tao, Jie Yang, Jian Su. **Suzhou CDC:** Fang liu, Jun Zhang, Yihe Hu, Yan Lu, , Liangcai Ma, Aiyu Tang, Shuo Zhang, Jianrong Jin, Jingchao Liu. **Guangxi Provincial CDC:** Zhenzhu Tang, Naying Chen, Ying Huang. **Liuzhou CDC:** Mingqiang Li, Jinhuai Meng, Rong Pan, Qilian Jiang, Jian Lan,Yun Liu, Liuping Wei, Liyuan Zhou, Ningyu Chen Ping Wang, Fanwen Meng, Yulu Qin,, Sisi Wang. **Sichuan Provincial CDC:** Xianping Wu, Ningmei Zhang, Xiaofang Chen,Weiwei Zhou. **Pengzhou CDC:** Guojin Luo, Jianguo Li, Xiaofang Chen, Xunfu Zhong, Jiaqiu Liu, Qiang Sun. **Gansu Provincial CDC:** Pengfei Ge, Xiaolan Ren, Caixia Dong. **Maiji CDC:** Hui Zhang, Enke Mao, Xiaoping Wang, Tao Wang, Xi zhang. **Henan Provincial CDC:** Ding Zhang, Gang Zhou, Shixian Feng, Liang Chang, Lei Fan. **Huixian CDC:** Yulian Gao, Tianyou He, Huarong Sun, Pan He, Chen Hu, Xukui Zhang, Huifang Wu, Pan He. **Zhejiang Provincial CDC:** Min Yu, Ruying Hu, Hao Wang. Tongxiang CDC: Yijian Qian, Chunmei Wang, Kaixu Xie, Lingli Chen, Yidan Zhang, Dongxia Pan, Qijun Gu. **Hunan Provincial CDC:** Yuelong Huang, Biyun Chen, Li Yin, Huilin Liu, Zhongxi Fu, Qiaohua Xu. **Liuyang CDC:** Xin Xu, Hao Zhang, Huajun Long, Xianzhi Li, Libo Zhang, Zhe Qiu.

Supplementary methods

Changes in LFs between baseline and resurvey

This analysis was based on the 25 038 participants who participated in the resurvey during 2013-14. We excluded participants who reported medical histories of heart disease (n=712), stroke (n=220), or cancer (n=100) at baseline. We also excluded those who had a self-reported history of diabetes or screen-detected diabetes, defined as measured fasting blood glucose ≥7.0 mmol/L or random blood glucose ≥11.1 mmol/L at baseline (n=1 041). After these exclusions, 22 965 participants remained in the present analysis.

We calculated the difference in total physical activity level (measured by the metabolic equivalent of task hours per day) between the 2004-08 baseline and 2013-14 resurvey. Other LFs were dichotomized based on the criteria of high-risk lifestyle in the primary analysis. Changes in LFs between baseline and resurvey were classified into stable (at the same risk level), better (from high-risk to low-risk lifestyle), and worse (from low-risk to high-risk lifestyle). The proportions (95% confidence intervals) of change in LFs and means (standard deviations) of difference in total physical activity by cardiometabolic disease status at 2013-14 resurvey were calculated using direct standardization to age in the 5-year interval, sex, and study area of CKB population, as appropriate.

Table S1. Food frequency questionnaire (FFQ) used in the CKB study at baseline

**During the past 12 months, about how often did you eat the following foods?**

|  | Daily | 4-6 days/week | 1-3 days/week | Monthly | Never/rarely |
| --- | --- | --- | --- | --- | --- |
| Rice | □ | □ | □ | □ | □ |
| Wheat | □ | □ | □ | □ | □ |
| Other staple food (corn, millet, etc.) | □ | □ | □ | □ | □ |
| Meat | □ | □ | □ | □ | □ |
| Poultry | □ | □ | □ | □ | □ |
| Fish/sea food | □ | □ | □ | □ | □ |
| Fresh eggs | □ | □ | □ | □ | □ |
| Fresh vegetables | □ | □ | □ | □ | □ |
| Soybean products | □ | □ | □ | □ | □ |
| Preserved vegetables | □ | □ | □ | □ | □ |
| Fresh fruit | □ | □ | □ | □ | □ |
| Dairy products (milk, yoghurt) | □ | □ | □ | □ | □ |

Table S2. Baseline characteristics of 189 099 men by incident disease status during follow-up

|  | CMD free | FCMD survivor | CMM survivor | Dead without CMD | Dead with FCMD | Dead with CMM | Total |
| --- | --- | --- | --- | --- | --- | --- | --- |
| No. of participants, n | 139 451 | 23 665 | 4 219 | 11 788 | 8 284 | 1 692 | 189 099 |
| Age at baseline, year | 49.7(9.9) | 55.6(10.0) | 59.0(9.5) | 60.5(10.3) | 63.5(9.3) | 65.2(8.2) | 52.1(10.8) |
| Urban, % | 42.2 | 44.4 | 52.7 | 32.7 | 29.1 | 38.1 | 41.5 |
| Middle school and above, % | 58.0 | 57.9 | 60.0 | 52.3 | 53.7 | 53.8 | 57.5 |
| Married, % | 93.7 | 93.7 | 94.5 | 89.3 | 88.6 | 91.3 | 93.0 |
| Parental family history of CMM, % | 0.8 | 1.1 | 1.2 | 0.7 | 0.9 | 0.5 | 0.9 |
| Prevalent hypertension, % | 30.8 | 45.9 | 54.8 | 34.8 | 52.7 | 57.9 | 34.8 |
| Dietary metrics, % |  |  |  |  |  |  |  |
| Eating vegetable daily | 94.9 | 94.7 | 95.6 | 94.0 | 94.3 | 93.5 | 94.8 |
| Eating fruit daily | 14.0 | 13.8 | 14.2 | 11.8 | 12.3 | 13.9 | 13.8 |
| Eating meat 1-6 days per week | 64.7 | 62.8 | 61.3 | 64.3 | 62.5 | 62.9 | 64.3 |
| Eating egg daily | 14.4 | 13.8 | 13.3 | 14.1 | 12.2 | 14.3 | 14.1 |
| Having high-risk lifestyle^a^, % |  |  |  |  |  |  |  |
| Tobacco smoking | 66.9 | 69.7 | 68.9 | 75.0 | 73.1 | 72.7 | 68.1 |
| Excessive alcohol drinking | 23.2 | 25.7 | 26.9 | 28.7 | 27.0 | 27.4 | 24.1 |
| Less healthy dietary habits | 98.6 | 98.7 | 98.7 | 98.9 | 99.0 | 98.9 | 98.7 |
| Low physical activity | 49.0 | 51.7 | 54.3 | 52.6 | 55.8 | 55.5 | 50.0 |
| Unhealthy body shape | 23.5 | 31.9 | 38.2 | 28.3 | 31.0 | 34.5 | 25.7 |
| Number of high-risk lifestyle factors, % |  |  |  |  |  |  |  |
| 0-1 | 10.7 | 8.5 | 7.9 | 6.9 | 7.1 | 6.7 | 9.9 |
| 2 | 36.2 | 31.5 | 29.1 | 30.2 | 29.4 | 28.2 | 34.7 |
| 3 | 36.3 | 37.4 | 36.8 | 38.4 | 37.8 | 38.4 | 36.7 |
| 4 | 14.7 | 18.9 | 21.2 | 20.8 | 21.5 | 22.5 | 16.1 |
| 5 | 2.1 | 3.7 | 5.1 | 3.7 | 4.3 | 4.2 | 2.6 |

CMD, cardiometabolic diseases; FCMD, first cardiometabolic disease; CMM, cardiometabolic multimorbidity.

Cardiometabolic diseases include ischemic heart disease, stroke, and type 2 diabetes. Cardiometabolic multimorbidity is defined as occurring at least two of the above-mentioned diseases. All variables were adjusted for age at enrollment and study areas, as appropriate. Results are given as a percentage or mean (standard deviation), as appropriate.

^a^High-risk lifestyle factors were defined as follows: current smoking or having stopped because of illness; daily drinking ≥30g/d of pure alcohol or having stopped drinking habit; non-daily eating of vegetables, fruits, and eggs, and eating red meat daily or less than weekly; engaging in a sex- and age-specific lower half of total physical activity; having BMI <18.5 or ≥28.0 kg/m^2^ or having waist circumference ≥90cm (men) / 85cm (women).

Table S3. Baseline characteristics of 271 948 women by incident disease status during follow-up

|  | CMD free | FCMD survivor | CMM survivor | Dead without CMD | Dead with FCMD | Dead with CMM | Total |
| --- | --- | --- | --- | --- | --- | --- | --- |
| No. of participants, n | 214 057 | 35 501 | 6 761 | 8 064 | 6 073 | 1 492 | 271 948 |
| Age at baseline, year | 48.8(9.6) | 55.0(9.6) | 58.4(8.8) | 59.3(10.8) | 63.4(9.3) | 65.6(7.8) | 50.6(10.3) |
| Urban, % | 43.2 | 44.4 | 51.9 | 33.7 | 25.7 | 32.6 | 42.8 |
| Middle school and above, % | 44.1 | 42.9 | 43.7 | 39.8 | 36.5 | 36.2 | 43.7 |
| Married, % | 89.8 | 90.6 | 90.9 | 87.7 | 87.1 | 88.2 | 89.8 |
| Parental family history of CMM, % | 0.9 | 1.2 | 1.2 | 0.8 | 0.7 | 0.8 | 1.0 |
| Prevalent hypertension, % | 27.9 | 39.9 | 45.5 | 29.4 | 45.0 | 48.8 | 30.6 |
| Dietary metrics, % |  |  |  |  |  |  |  |
| Eating vegetable daily | 94.5 | 94.9 | 95.4 | 93.8 | 93.4 | 94.8 | 94.5 |
| Eating fruit daily | 21.4 | 21.1 | 21.8 | 19.3 | 17.3 | 19.8 | 21.2 |
| Eating meat 1-6 days per week | 68.6 | 67.3 | 66.2 | 68.2 | 66.6 | 67.2 | 68.3 |
| Eating egg daily | 12.5 | 12.0 | 11.9 | 12.2 | 10.7 | 11.6 | 12.4 |
| Having high-risk lifestyle^a^, % |  |  |  |  |  |  |  |
| Tobacco smoking | 2.5 | 2.7 | 2.6 | 3.7 | 3.5 | 3.5 | 2.6 |
| Excessive alcohol drinking | 1.2 | 1.3 | 1.1 | 1.3 | 1.3 | 1.5 | 1.2 |
| Less healthy dietary habits | 97.6 | 97.8 | 97.9 | 97.9 | 98.3 | 98.5 | 97.7 |
| Low physical activity | 49.4 | 50.6 | 52.7 | 55.2 | 59.7 | 58.0 | 50.1 |
| Unhealthy body shape | 27.8 | 35.2 | 39.9 | 28.7 | 31.7 | 37.2 | 29.3 |
| Number of high-risk lifestyle factors, % |  |  |  |  |  |  |  |
| 0-1 | 37.6 | 32.7 | 29.6 | 32.4 | 28.9 | 26.8 | 36.4 |
| 2 | 46.4 | 47.3 | 47.7 | 48.4 | 48.7 | 48.6 | 46.5 |
| 3 | 15.4 | 19.3 | 21.7 | 18.1 | 21.1 | 23.1 | 16.4 |
| 4 | 0.6 | 0.8 | 0.9 | 1.0 | 1.2 | 1.4 | 0.7 |
| 5 | <0.1 | <0.1 | 0.1 | 0.1 | 0.1 | 0.1 | <0.1 |

CMD, cardiometabolic diseases; FCMD, first cardiometabolic disease; CMM, cardiometabolic multimorbidity.

Cardiometabolic diseases include ischemic heart disease, stroke and type 2 diabetes. Cardiometabolic multimorbidity is defined as occurring at least two of the above-mentioned diseases.

All variables were adjusted for age at enrollment and study areas, as appropriate. Results are given as a percentage or mean (standard deviation), as appropriate.

^a^High-risk lifestyle factors were defined as follows: current smoking or having stopped because of illness; daily drinking ≥30g/d of pure alcohol or having stopped drinking habit; non-daily eating of vegetables, fruits, and eggs, and eating red meat daily or less than weekly; engaging in a sex- and age-specific lower half of total physical activity; having BMI <18.5 or ≥28.0 kg/m^2^ or having waist circumference ≥90cm (men) / 85cm (women).

Table S4. Changes in lifestyle factors between 2004-08 baseline and 2013-14 resurvey by cardiometabolic disease status at 2013-14 resurvey among 22 965 participants

|  | None | One CMD | CMM | Total |
| --- | --- | --- | --- | --- |
| No. of participants, n | 20 699 | 2 065 | 201 | 22 965 |
| Age at baseline, years | 50.3 (9.9) | 56.2 (9.6) | 59.0 (8.1) | 50.9 (10.0) |
| Age at resurvey, years | 58.3 (9.9) | 64.3 (9.6) | 67.1 (8.1) | 58.9 (10.1) |
| Physical activity, MET-h/d^a^ | -3.3 (16.4) | -4.3 (15.0) | -3.7 (11.9) | -3.4 (16.2) |
| Smoking |  |  |  |  |
| Stable | 93.3 (92.9-93.6) | 91.9 (90.8-92.9) | 90.9 (87.4-94.3) | 93.1 (92.8-93.4) |
| Worse | 1.5 (1.3-1.6) | 1.4 (0.9-1.8) | 0.9 (-0.3-2.1) | 1.5 (0.5-3.5) |
| Better | 5.3 (5.0-5.6) | 6.8 (5.8-7.8) | 8.2 (4.9-11.6) | 5.4 (4.2-6.8) |
| Alcohol drinking |  |  |  |  |
| Stable | 90.8 (90.4-91.2) | 89.3 (88.1-90.6) | 84.3 (79.9-88.8) | 90.6 (90.2-91.0) |
| Worse | 5.1 (4.8-5.4) | 6.0 (5.0-6.9) | 8.4 (4.8-12.0) | 5.3 (4.1-6.7) |
| Better | 4.1 (3.8-4.4) | 4.7 (3.8-5.6) | 7.3 (3.9-10.6) | 4.1 (3.0-5.6) |
| Dietary habits |  |  |  |  |
| Stable | 94.4 (94.1-94.7) | 94.7 (93.8-95.6) | 95.5 (93.1-98.0) | 94.4 (94.1-94.7) |
| Worse | 1.3 (1.1-1.4) | 1.1 (0.7-1.5) | 2.5 (0.7-4.3) | 1.4 (0.4-3.5) |
| Better | 4.3 (4.1-4.6) | 4.2 (3.4-5.0) | 2.0 (0.3-3.7) | 4.4 (3.2-5.8) |
| Body shape |  |  |  |  |
| Stable | 73.2 (72.6-73.8) | 75.6 (73.8-77.5) | 75.5 (69.7-81.4) | 73.4 (72.8-74.1) |
| Worse | 22.6 (22.0-23.1) | 20.6 (18.9-22.4) | 21.5 (15.9-27.1) | 22.4 (21.3-23.6) |
| Better | 4.2 (4.0-4.5) | 3.7 (2.9-4.5) | 3.0 (0.8-5.1) | 4.2 (3.0-5.6) |

CMD, cardiometabolic disease; CMM, cardiometabolic multimorbidity; MET-h/d, metabolic equivalent of task hours per day.

Cardiometabolic diseases include ischemic heart disease, stroke, and type 2 diabetes. Cardiometabolic multimorbidity is defined as occurring two of the above-mentioned diseases.

All variables were adjusted for age at enrollment, sex, and study area, as appropriate. Age and physical activity are given means (standard deviations). The others are given as percentages (95% confidence intervals).

^a^Difference in physical activity between baseline and resurvey.

Other lifestyle factors were dichotomized according to the following criteria: current smoking or having stopped because of illness; daily drinking ≥30g/d of pure alcohol or having stopped drinking habit; non-daily eating of vegetables, fruits, and eggs, and eating red meat daily or less than weekly; having BMI <18.5 or ≥28.0 kg/m^2^ or having waist circumference ≥90cm (men) / 85cm (women).

Changes in lifestyle factors between baseline and resurvey were classified into stable (at the same risk level), better (from high-risk to low-risk lifestyle), and worse (from low-risk to high-risk lifestyle).

Table S5. HRs (95% CIs) for incident first cardiometabolic disease, cardiometabolic multimorbidity, and all-cause mortality by lifestyle factors among 461 047 participants

|  | First cardiometabolic disease | | |  | Cardiometabolic multimorbidity | | |  | Death | | |
| --- | --- | --- | --- | --- | --- | --- | --- | --- | --- | --- | --- |
|  | Cases | Cases/PYs | HRs (95% CIs) |  | Cases | Cases/PYs | HRs (95% CIs) |  | Cases | Cases/PYs | HRs (95% CIs) |
|  |  | (1/10,000) |  |  |  | (1/10,000) |  |  |  | (1/10,000) |  |
| Tobacco smoking | |  |  |  |  |  |  |  |  |  |  |
| No | 59 721 | 179.67 | Reference |  | 9 794 | 27.61 | Reference |  | 20 368 | 56.96 | Reference |
| Yes | 27 966 | 207.09 | 1.13 (1.11-1.16) | | 4 370 | 30.24 | 1.16 (1.11-1.22) | | 17 025 | 116.84 | 1.35 (1.31-1.38) |
| Excessive alcohol drinking | | |  |  |  |  |  |  |  |  |  |
| No | 77 163 | 183.98 | Reference |  | 12 392 | 27.69 | Reference |  | 30 437 | 67.46 | Reference |
| Yes | 10 524 | 219.06 | 1.10 (1.07-1.12) | | 1 772 | 34.34 | 1.16 (1.09-1.22) | | 6 956 | 133.56 | 1.24 (1.21-1.28) |
| Less healthy dietary habits | | |  |  |  |  |  |  |  |  |  |
| No | 1 976 | 224.95 | Reference |  | 403 | 42.41 | Reference |  | 576 | 59.83 | Reference |
| Yes | 85 711 | 186.87 | 1.13 (1.08-1.18) | | 13 761 | 28.10 | 1.18 (1.06-1.30) | | 36 817 | 74.58 | 1.12 (1.03-1.22) |
| Low physical activity | |  |  |  |  |  |  |  |  |  |  |
| No | 38 881 | 164.16 | Reference |  | 5 591 | 22.25 | Reference |  | 16 739 | 66.21 | Reference |
| Yes | 48 806 | 211.65 | 1.09 (1.08-1.11) | | 8 573 | 34.57 | 1.14 (1.10-1.18) | | 20 654 | 82.46 | 1.21 (1.18-1.23) |
| Unhealthy body shape | | |  |  |  |  |  |  |  |  |  |
| No | 54 779 | 159.49 | Reference |  | 7 962 | 21.91 | Reference |  | 25 118 | 68.70 | Reference |
| Yes | 32 908 | 265.41 | 1.40 (1.38-1.42) | | 6 202 | 45.66 | 1.63 (1.57-1.68) | | 12 275 | 89.16 | 1.17 (1.14-1.19) |
| Number of high-risk lifestyle factors | | | |  |  |  |  |  |  |  |  |
| 0-1 | 16 637 | 134.38 | Reference |  | 2 224 | 17.07 | Reference |  | 5 620 | 42.93 | Reference |
| 2 | 34 939 | 178.00 | 1.17 (1.15-1.19) | | 5 481 | 26.22 | 1.29 (1.22-1.35) | | 13 739 | 65.22 | 1.18 (1.14-1.22) |
| 3 | 26 304 | 234.97 | 1.41 (1.38-1.44) | | 4 617 | 38.10 | 1.66 (1.58-1.76) | | 12 313 | 100.50 | 1.44 (1.39-1.49) |
| 4 | 8 219 | 266.84 | 1.70 (1.65-1.75) | | 1 513 | 45.10 | 2.14 (1.99-2.30) | | 4 845 | 142.68 | 1.86 (1.79-1.94) |
| 5 | 1 588 | 344.95 | 2.16 (2.05-2.28) | | 329 | 63.92 | 2.96 (2.63-3.34) | | 876 | 167.10 | 2.21 (2.05-2.38) |
| Per 1-factor increase | |  | 1.20 (1.19-1.21) | |  |  | 1.29 (1.27-1.32) | |  |  | 1.23 (1.21-1.24) |

HR, hazard ratio; CI, confidence interval; PYs, person-years.

Cardiometabolic diseases include ischemic heart disease, stroke, and type 2 diabetes. Cardiometabolic multimorbidity is defined as occurring at least two of the above-mentioned diseases. Multivariable models were stratified by age in the 5-year interval and study areas, and adjusted for sex, education, marital status, and parental family history of cardiometabolic multimorbidity. For analyses of dichotomous lifestyle factors, five lifestyle factors were mutually adjusted.

^a^High-risk lifestyle factors were defined as follows: current smoking or having stopped because of illness; daily drinking ≥30g/d of pure alcohol or having stopped drinking habit; non-daily eating of vegetables, fruits, and eggs, and eating red meat daily or less than weekly; engaging in a sex- and age-specific lower half of total physical activity; having BMI <18.5 or ≥28.0 kg/m^2^ or having waist circumference ≥90cm (men) / 85cm (women).

Table S6. Sensitivity analysis of associations of a 1-factor increase in the high-risk lifestyle factors with transitions of cardiometabolic diseases among 461 047 participants

|  | HRs (95%CIs) | | | | | |
| --- | --- | --- | --- | --- | --- | --- |
|  | Baseline → FCMD | FCMD → CMM | Baseline → Death | FCMD → Death | CMM → Death | Baseline → CMM |
| **Tobacco smoking** |  |  |  |  |  |  |
| Different interval |  |  |  |  |  |  |
| 0.5 days | 1.13 (1.11-1.16) | 1.07 (1.02-1.12) | 1.40 (1.34-1.45) | 1.20 (1.14-1.25) | 1.27 (1.16-1.40) |  |
| 0.5 years | 1.13 (1.11-1.16) | 1.06 (1.01-1.12) | 1.40 (1.34-1.45) | 1.19 (1.14-1.24) | 1.27 (1.15-1.40) |  |
| 1 year | 1.13 (1.11-1.16) | 1.06 (1.01-1.11) | 1.40 (1.34-1.45) | 1.20 (1.14-1.25) | 1.26 (1.15-1.39) |  |
| 3 years | 1.13 (1.11-1.16) | 1.06 (1.01-1.11) | 1.40 (1.35-1.45) | 1.21 (1.15-1.26) | 1.30 (1.18-1.43) |  |
| 5 years | 1.13 (1.11-1.15) | 1.05 (1.00-1.11) | 1.40 (1.35-1.45) | 1.21 (1.15-1.27) | 1.32 (1.18-1.48) |  |
| Excluding participants who entered different states on the same date | 1.13 (1.11-1.15) | 1.07 (1.01-1.12) | 1.40 (1.35-1.45) | 1.26 (1.19-1.34) | 1.38 (1.22-1.57) |  |
| Additional adjustment for hypertension, usage of blood pressure medication and statin | 1.17 (1.15-1.20) | 1.08 (1.03-1.13) | 1.40 (1.34-1.45) | 1.21 (1.16-1.27) | 1.29 (1.17-1.42) |  |
| Including baseline cardiometabolic patients | 1.13 (1.11-1.16) | 1.09 (1.05-1.13) | 1.39 (1.34-1.45) | 1.29 (1.25-1.34) | 1.23 (1.15-1.30) |  |
| Excluding events occurred in the first two-year of follow-up | 1.15 (1.12-1.17) | 1.06 (1.00-1.12) | 1.41 (1.35-1.47) | 1.23 (1.17-1.29) | 1.16 (1.05-1.30) |  |
| Adding a transition from healthy directly to CMM | 1.13 (1.11-1.16) | 1.07 (1.02-1.13) | 1.40 (1.34-1.45) | 1.20 (1.14-1.25) | 1.27 (1.16-1.40) | 1.13 (0.97-1.31) |
| **Excessive alcohol drinking** |  |  |  |  |  |  |
| Different interval |  |  |  |  |  |  |
| 0.5 days | 1.10 (1.07-1.12) | 1.07 (1.01-1.13) | 1.27 (1.22-1.32) | 1.14 (1.09-1.20) | 1.12 (1.00-1.24) |  |
| 0.5 years | 1.10 (1.07-1.12) | 1.07 (1.01-1.13) | 1.27 (1.22-1.32) | 1.15 (1.09-1.20) | 1.12 (1.01-1.25) |  |
| 1 year | 1.10 (1.07-1.12) | 1.06 (1.01-1.13) | 1.27 (1.22-1.32) | 1.15 (1.09-1.21) | 1.11 (1.00-1.24) |  |
| 3 years | 1.10 (1.07-1.12) | 1.06 (1.00-1.12) | 1.27 (1.22-1.32) | 1.16 (1.10-1.22) | 1.09 (0.98-1.22) |  |
| 5 years | 1.10 (1.07-1.12) | 1.07 (1.01-1.13) | 1.27 (1.22-1.32) | 1.17 (1.11-1.23) | 1.15 (1.02-1.30) |  |
| Excluding participants who entered different states on the same date | 1.09 (1.07-1.12) | 1.06 (1.00-1.13) | 1.27 (1.22-1.32) | 1.18 (1.11-1.25) | 1.17 (1.02-1.34) |  |
| Additional adjustment for hypertension, usage of blood pressure medication and statin | 1.05 (1.03-1.07) | 1.05 (0.99-1.11) | 1.27 (1.22-1.32) | 1.13 (1.07-1.18) | 1.09 (0.98-1.22) |  |
| Including baseline cardiometabolic patients | 1.10 (1.07-1.12) | 1.01 (0.97-1.06) | 1.27 (1.22-1.32) | 1.13 (1.08-1.17) | 1.01 (0.94-1.09) |  |
| Excluding events occurred in the first two-year of follow-up | 1.10 (1.07-1.12) | 1.07 (1.01-1.14) | 1.27 (1.22-1.32) | 1.13 (1.07-1.19) | 1.10 (0.97-1.24) |  |
| Adding a transition from healthy directly to CMM | 1.10 (1.07-1.12) | 1.07 (1.01-1.13) | 1.27 (1.22-1.32) | 1.14 (1.09-1.20) | 1.12 (1.00-1.24) | 1.18 (0.99-1.40) |
| **Less healthy dietary habits** |  |  |  |  |  |  |
| Different interval |  |  |  |  |  |  |
| 0.5 days | 1.13 (1.08-1.18) | 1.04 (0.94-1.16) | 1.02 (0.91-1.14) | 1.13 (0.98-1.31) | 1.17 (0.89-1.53) |  |
| 0.5 years | 1.12 (1.07-1.18) | 1.04 (0.94-1.15) | 1.02 (0.91-1.14) | 1.13 (0.98-1.31) | 1.17 (0.89-1.53) |  |
| 1 year | 1.12 (1.07-1.18) | 1.05 (0.94-1.16) | 1.02 (0.91-1.14) | 1.12 (0.97-1.30) | 1.16 (0.89-1.53) |  |
| 3 years | 1.12 (1.07-1.17) | 1.04 (0.94-1.15) | 1.02 (0.91-1.15) | 1.15 (0.99-1.33) | 1.12 (0.85-1.48) |  |
| 5 years | 1.12 (1.07-1.17) | 1.04 (0.94-1.15) | 1.02 (0.91-1.15) | 1.16 (0.99-1.35) | 1.19 (0.87-1.61) |  |
| Excluding participants who entered different states on the same date | 1.13 (1.08-1.18) | 1.06 (0.95-1.18) | 1.02 (0.91-1.14) | 1.20 (1.00-1.44) | 1.18 (0.84-1.66) |  |
| Additional adjustment for hypertension, usage of blood pressure medication and statin | 1.12 (1.07-1.17) | 1.04 (0.94-1.15) | 1.02 (0.91-1.14) | 1.12 (0.97-1.29) | 1.17 (0.89-1.54) |  |
| Including baseline cardiometabolic patients | 1.13 (1.08-1.18) | 1.14 (1.06-1.23) | 1.02 (0.91-1.14) | 1.20 (1.08-1.33) | 1.06 (0.93-1.22) |  |
| Excluding events occurred in the first two-year of follow-up | 1.12 (1.06-1.17) | 1.05 (0.94-1.18) | 1.00 (0.89-1.13) | 1.12 (0.96-1.31) | 1.05 (0.77-1.42) |  |
| Adding a transition from healthy directly to CMM | 1.13 (1.08-1.18) | 1.06 (0.95-1.18) | 1.02 (0.91-1.14) | 1.13 (0.98-1.31) | 1.17 (0.89-1.53) | 1.02 (0.74-1.39) |
| **Low physical activity** |  |  |  |  |  |  |
| Different interval |  |  |  |  |  |  |
| 0.5 days | 1.09 (1.08-1.11) | 1.08 (1.05-1.12) | 1.17 (1.13-1.21) | 1.22 (1.18-1.27) | 1.07 (0.99-1.15) |  |
| 0.5 years | 1.09 (1.08-1.11) | 1.08 (1.04-1.12) | 1.17 (1.13-1.21) | 1.21 (1.16-1.25) | 1.08 (1.00-1.17) |  |
| 1 year | 1.09 (1.08-1.11) | 1.08 (1.04-1.12) | 1.17 (1.14-1.21) | 1.19 (1.15-1.24) | 1.09 (1.01-1.18) |  |
| 3 years | 1.09 (1.08-1.11) | 1.08 (1.04-1.12) | 1.17 (1.14-1.21) | 1.17 (1.12-1.21) | 1.09 (1.00-1.18) |  |
| 5 years | 1.09 (1.07-1.10) | 1.08 (1.04-1.12) | 1.17 (1.14-1.21) | 1.16 (1.11-1.20) | 1.10 (1.01-1.21) |  |
| Excluding participants who entered different states on the same date | 1.08 (1.07-1.10) | 1.10 (1.06-1.15) | 1.18 (1.14-1.21) | 1.18 (1.12-1.23) | 1.17 (1.05-1.29) |  |
| Additional adjustment for hypertension, usage of blood pressure medication and statin | 1.07 (1.06-1.09) | 1.07 (1.04-1.11) | 1.17 (1.14-1.21) | 1.21 (1.17-1.26) | 1.06 (0.98-1.14) |  |
| Including baseline cardiometabolic patients | 1.10 (1.08-1.11) | 1.06 (1.03-1.09) | 1.17 (1.13-1.20) | 1.18 (1.15-1.22) | 1.10 (1.05-1.16) |  |
| Excluding events occurred in the first two-year of follow-up | 1.08 (1.06-1.09) | 1.08 (1.04-1.13) | 1.12 (1.08-1.16) | 1.21 (1.16-1.26) | 1.05 (0.96-1.15) |  |
| Adding a transition from healthy directly to CMM | 1.09 (1.08-1.11) | 1.09 (1.05-1.13) | 1.17 (1.13-1.21) | 1.22 (1.18-1.27) | 1.07 (0.99-1.15) | 1.10 (0.98-1.23) |
| **Unhealthy body shape** |  |  |  |  |  |  |
| Different interval |  |  |  |  |  |  |
| 0.5 days | 1.40 (1.38-1.42) | 1.27 (1.23-1.31) | 1.11 (1.08-1.15) | 0.99 (0.95-1.02) | 1.03 (0.95-1.11) |  |
| 0.5 years | 1.40 (1.38-1.42) | 1.27 (1.22-1.31) | 1.11 (1.08-1.15) | 0.99 (0.95-1.03) | 1.02 (0.95-1.10) |  |
| 1 year | 1.40 (1.38-1.42) | 1.27 (1.22-1.31) | 1.11 (1.08-1.15) | 0.99 (0.95-1.02) | 1.02 (0.95-1.10) |  |
| 3 years | 1.41 (1.39-1.43) | 1.27 (1.23-1.31) | 1.11 (1.08-1.15) | 0.99 (0.95-1.03) | 1.05 (0.97-1.13) |  |
| 5 years | 1.41 (1.39-1.43) | 1.28 (1.23-1.32) | 1.12 (1.08-1.15) | 0.99 (0.96-1.03) | 1.10 (1.01-1.20) |  |
| Excluding participants who entered different states on the same date | 1.42 (1.40-1.44) | 1.28 (1.23-1.33) | 1.12 (1.08-1.15) | 1.00 (0.95-1.05) | 1.09 (0.99-1.20) |  |
| Additional adjustment for hypertension, usage of blood pressure medication and statin | 1.29 (1.28-1.31) | 1.22 (1.18-1.26) | 1.11 (1.08-1.15) | 0.97 (0.94-1.01) | 1.01 (0.94-1.09) |  |
| Including baseline cardiometabolic patients | 1.40 (1.38-1.42) | 1.22 (1.19-1.25) | 1.11 (1.08-1.15) | 0.95 (0.93-0.98) | 1.01 (0.97-1.06) |  |
| Excluding events occurred in the first two-year of follow-up | 1.41 (1.39-1.43) | 1.26 (1.21-1.31) | 1.09 (1.05-1.13) | 0.97 (0.93-1.01) | 1.04 (0.96-1.14) |  |
| Adding a transition from healthy directly to CMM | 1.40 (1.38-1.42) | 1.26 (1.21-1.30) | 1.11 (1.08-1.15) | 0.99 (0.95-1.02) | 1.03 (0.95-1.11) | 1.89 (1.70-2.11) |

HR, hazard ratio; CI, confidence interval; CMD, cardiometabolic disease; FCMD, first cardiometabolic disease; CMM, cardiometabolic multimorbidity.

Cardiometabolic diseases include ischemic heart disease, stroke, and type 2 diabetes. Cardiometabolic multimorbidity is defined as occurring at least two of the above-mentioned diseases.

Multivariable models were stratified by age in the 5-year interval, region area, and transition, and adjusted for sex, education, marital status, family history of CMM, as appropriate. (1) calculating the entering date of the prior state using different time intervals (0.5-day, 0.5-year, 1-year, 3-year, 5-year) for participants who entered different states on the same day; (2) excluding participants who entered different states on the same date; (3) additionally adjusting for hypertension, usage of blood pressure medicine and statin at baseline; (4) including participants who had previously diagnosed heart disease, stroke, or diabetes, and assigning them to FCMD or CMM state according to their CMD status at baseline; (5) excluding the events occurring in the first two years follow-up; (6) in addition to the predefined five transitions, adding another transition from the baseline directly to CMM.

High-risk lifestyle factors were defined as follows: current smoking or having stopped because of illness; daily drinking ≥30g/d of pure alcohol or having stopped drinking habit; non-daily eating of vegetables, fruits, and eggs, and eating red meat daily or less than weekly; engaging in a sex- and age-specific lower half of total physical activity; having BMI <18.5 or ≥28.0 kg/m^2^ or having waist circumference ≥90cm (men) / 85cm (women).

Table S7. Sensitivity analysis of associations of the number of high-risk lifestyle factors with transitions of cardiometabolic diseases among 461 047 participants

|  | HRs (95%CIs) | | | | | |
| --- | --- | --- | --- | --- | --- | --- |
|  | Baseline → FCMD | FCMD → CMM | Baseline → Death | FCMD → Death | CMM → Death | Baseline → CMM |
| **0-1** | Reference | Reference | Reference | Reference | Reference | Reference |
| **2** |  |  |  |  |  |  |
| Different interval |  |  |  |  |  |  |
| 0.5 days | 1.17 (1.15-1.19) | 1.16 (1.10-1.22) | 1.17 (1.12-1.22) | 1.09 (1.03-1.15) | 1.08 (0.96-1.22) |  |
| 0.5 years | 1.17 (1.15-1.19) | 1.16 (1.10-1.22) | 1.17 (1.12-1.22) | 1.09 (1.03-1.14) | 1.09 (0.96-1.22) |  |
| 1 year | 1.17 (1.15-1.19) | 1.16 (1.10-1.22) | 1.17 (1.12-1.22) | 1.08 (1.03-1.14) | 1.09 (0.97-1.23) |  |
| 3 years | 1.17 (1.15-1.19) | 1.16 (1.10-1.22) | 1.17 (1.12-1.22) | 1.09 (1.03-1.15) | 1.11 (0.98-1.25) |  |
| 5 years | 1.17 (1.15-1.19) | 1.17 (1.11-1.23) | 1.17 (1.12-1.22) | 1.08 (1.02-1.15) | 1.18 (1.02-1.36) |  |
| Excluding participants who entered different states on the same date | 1.17 (1.15-1.19) | 1.19 (1.13-1.26) | 1.17 (1.12-1.22) | 1.09 (1.02-1.17) | 1.21 (1.03-1.43) |  |
| Additional adjustment for hypertension, usage of blood pressure medication and statin | 1.13 (1.11-1.15) | 1.14 (1.09-1.20) | 1.17 (1.12-1.22) | 1.08 (1.03-1.14) | 1.08 (0.96-1.21) |  |
| Including baseline cardiometabolic patients | 1.17 (1.15-1.19) | 1.13 (1.09-1.18) | 1.18 (1.13-1.23) | 1.09 (1.04-1.14) | 1.08 (1.00-1.17) |  |
| Excluding events occurred in the first two-year of follow-up | 1.16 (1.14-1.19) | 1.15 (1.09-1.21) | 1.13 (1.08-1.18) | 1.08 (1.02-1.15) | 1.04 (0.92-1.19) |  |
| Adding a transition from healthy directly to CMM | 1.17 (1.14-1.19) | 1.17 (1.11-1.23) | 1.17 (1.12-1.22) | 1.09 (1.03-1.15) | 1.08 (0.96-1.22) | 1.23 (1.05-1.44) |
| **3** |  |  |  |  |  |  |
| Different interval |  |  |  |  |  |  |
| 0.5 days | 1.41 (1.38-1.44) | 1.31 (1.24-1.38) | 1.38 (1.32-1.45) | 1.23 (1.16-1.29) | 1.23 (1.09-1.39) |  |
| 0.5 years | 1.41 (1.38-1.44) | 1.30 (1.23-1.37) | 1.39 (1.32-1.45) | 1.22 (1.15-1.28) | 1.23 (1.09-1.39) |  |
| 1 year | 1.41 (1.38-1.44) | 1.30 (1.23-1.37) | 1.39 (1.32-1.45) | 1.21 (1.15-1.28) | 1.23 (1.09-1.39) |  |
| 3 years | 1.41 (1.38-1.44) | 1.30 (1.23-1.37) | 1.39 (1.33-1.45) | 1.20 (1.13-1.26) | 1.24 (1.09-1.41) |  |
| 5 years | 1.41 (1.38-1.44) | 1.31 (1.24-1.39) | 1.39 (1.33-1.46) | 1.19 (1.12-1.26) | 1.35 (1.17-1.56) |  |
| Excluding participants who entered different states on the same date | 1.41 (1.38-1.44) | 1.34 (1.27-1.42) | 1.39 (1.33-1.46) | 1.22 (1.13-1.31) | 1.44 (1.22-1.70) |  |
| Additional adjustment for hypertension, usage of blood pressure medication and statin | 1.32 (1.29-1.34) | 1.26 (1.20-1.33) | 1.39 (1.33-1.45) | 1.21 (1.15-1.28) | 1.21 (1.07-1.37) |  |
| Including baseline cardiometabolic patients | 1.41 (1.38-1.44) | 1.27 (1.21-1.32) | 1.39 (1.33-1.45) | 1.20 (1.14-1.25) | 1.18 (1.09-1.28) |  |
| Excluding events occurred in the first two-year of follow-up | 1.40 (1.37-1.43) | 1.29 (1.22-1.37) | 1.32 (1.26-1.39) | 1.20 (1.13-1.28) | 1.17 (1.02-1.34) |  |
| Adding a transition from healthy directly to CMM | 1.41 (1.38-1.44) | 1.32 (1.24-1.39) | 1.38 (1.32-1.45) | 1.22 (1.16-1.29) | 1.23 (1.09-1.39) | 1.68 (1.42-1.98) |
| **4** |  |  |  |  |  |  |
| Different interval |  |  |  |  |  |  |
| 0.5 days | 1.70 (1.65-1.75) | 1.48 (1.37-1.59) | 1.81 (1.71-1.91) | 1.45 (1.35-1.55) | 1.43 (1.23-1.66) |  |
| 0.5 years | 1.70 (1.65-1.75) | 1.47 (1.37-1.58) | 1.81 (1.71-1.91) | 1.43 (1.33-1.53) | 1.44 (1.24-1.67) |  |
| 1 year | 1.70 (1.65-1.75) | 1.46 (1.36-1.57) | 1.81 (1.71-1.92) | 1.42 (1.32-1.52) | 1.45 (1.25-1.69) |  |
| 3 years | 1.70 (1.65-1.75) | 1.45 (1.35-1.56) | 1.82 (1.72-1.92) | 1.41 (1.31-1.51) | 1.50 (1.28-1.75) |  |
| 5 years | 1.70 (1.65-1.75) | 1.47 (1.37-1.59) | 1.82 (1.72-1.93) | 1.41 (1.31-1.52) | 1.69 (1.42-2.02) |  |
| Excluding participants who entered different states on the same date | 1.70 (1.65-1.75) | 1.51 (1.40-1.64) | 1.83 (1.72-1.93) | 1.50 (1.37-1.64) | 1.88 (1.54-2.29) |  |
| Additional adjustment for hypertension, usage of blood pressure medication and statin | 1.55 (1.50-1.59) | 1.40 (1.31-1.51) | 1.81 (1.71-1.92) | 1.42 (1.32-1.52) | 1.40 (1.20-1.63) |  |
| Including baseline cardiometabolic patients | 1.71 (1.66-1.76) | 1.38 (1.30-1.46) | 1.82 (1.72-1.93) | 1.42 (1.34-1.50) | 1.30 (1.18-1.44) |  |
| Excluding events occurred in the first two-year of follow-up | 1.69 (1.64-1.75) | 1.45 (1.34-1.57) | 1.70 (1.60-1.80) | 1.42 (1.32-1.53) | 1.32 (1.11-1.56) |  |
| Adding a transition from healthy directly to CMM | 1.69 (1.64-1.74) | 1.48 (1.37-1.59) | 1.81 (1.71-1.91) | 1.45 (1.35-1.55) | 1.43 (1.23-1.66) | 2.37 (1.90-2.96) |
| **5** |  |  |  |  |  |  |
| Different interval |  |  |  |  |  |  |
| 0.5 days | 2.16 (2.05-2.28) | 1.67 (1.48-1.88) | 2.12 (1.91-2.34) | 1.51 (1.34-1.70) | 1.17 (0.91-1.51) |  |
| 0.5 years | 2.16 (2.05-2.28) | 1.66 (1.47-1.87) | 2.12 (1.91-2.35) | 1.50 (1.33-1.69) | 1.17 (0.91-1.51) |  |
| 1 year | 2.16 (2.05-2.28) | 1.65 (1.46-1.87) | 2.12 (1.92-2.35) | 1.49 (1.32-1.68) | 1.18 (0.91-1.52) |  |
| 3 years | 2.17 (2.05-2.29) | 1.65 (1.46-1.87) | 2.13 (1.93-2.36) | 1.52 (1.34-1.72) | 1.27 (0.98-1.66) |  |
| 5 years | 2.17 (2.06-2.30) | 1.68 (1.49-1.91) | 2.14 (1.93-2.37) | 1.55 (1.36-1.76) | 1.43 (1.07-1.92) |  |
| Excluding participants who entered different states on the same date | 2.18 (2.06-2.31) | 1.73 (1.51-1.97) | 2.15 (1.94-2.38) | 1.66 (1.43-1.92) | 1.46 (1.05-2.03) |  |
| Additional adjustment for hypertension, usage of blood pressure medication and statin | 1.86 (1.76-1.96) | 1.56 (1.38-1.76) | 2.13 (1.92-2.35) | 1.47 (1.31-1.66) | 1.14 (0.88-1.47) |  |
| Including baseline cardiometabolic patients | 2.19 (2.07-2.31) | 1.48 (1.35-1.62) | 2.10 (1.89-2.33) | 1.52 (1.39-1.66) | 1.25 (1.08-1.44) |  |
| Excluding events occurred in the first two-year of follow-up | 2.16 (2.04-2.29) | 1.70 (1.49-1.94) | 2.04 (1.82-2.27) | 1.48 (1.30-1.69) | 1.08 (0.81-1.44) |  |
| Adding a transition from healthy directly to CMM | 2.15 (2.04-2.27) | 1.68 (1.48-1.90) | 2.12 (1.91-2.34) | 1.51 (1.34-1.70) | 1.17 (0.91-1.51) | 3.22 (2.19-4.74) |
| **Per 1-factor increase** |  |  |  |  |  |  |
| Different interval |  |  |  |  |  |  |
| 0.5 days | 1.20 (1.19-1.21) | 1.14 (1.11-1.16) | 1.21 (1.19-1.23) | 1.12 (1.10-1.15) | 1.10 (1.06-1.15) |  |
| 0.5 years | 1.20 (1.19-1.21) | 1.13 (1.11-1.16) | 1.21 (1.19-1.23) | 1.12 (1.10-1.14) | 1.10 (1.06-1.15) |  |
| 1 year | 1.20 (1.19-1.21) | 1.13 (1.11-1.15) | 1.21 (1.19-1.23) | 1.12 (1.10-1.14) | 1.11 (1.06-1.15) |  |
| 3 years | 1.20 (1.19-1.21) | 1.13 (1.11-1.15) | 1.21 (1.19-1.23) | 1.12 (1.09-1.14) | 1.12 (1.07-1.16) |  |
| 5 years | 1.20 (1.19-1.21) | 1.14 (1.11-1.16) | 1.21 (1.19-1.23) | 1.12 (1.09-1.14) | 1.15 (1.10-1.21) |  |
| Excluding participants who entered different states on the same date | 1.20 (1.19-1.21) | 1.14 (1.12-1.17) | 1.21 (1.19-1.23) | 1.14 (1.11-1.17) | 1.18 (1.12-1.25) |  |
| Additional adjustment for hypertension, usage of blood pressure medication and statin | 1.16 (1.15-1.17) | 1.12 (1.09-1.14) | 1.21 (1.19-1.23) | 1.12 (1.10-1.14) | 1.09 (1.05-1.14) |  |
| Including baseline cardiometabolic patients | 1.20 (1.19-1.21) | 1.11 (1.10-1.13) | 1.21 (1.19-1.23) | 1.12 (1.10-1.13) | 1.08 (1.05-1.11) |  |
| Excluding events occurred in the first two-year of follow-up | 1.19 (1.18-1.20) | 1.13 (1.11-1.16) | 1.19 (1.17-1.21) | 1.12 (1.09-1.14) | 1.08 (1.03-1.13) |  |
| Adding a transition from healthy directly to CMM | 1.19 (1.19-1.20) | 1.14 (1.11-1.16) | 1.21 (1.19-1.23) | 1.12 (1.10-1.15) | 1.10 (1.06-1.15) | 1.33 (1.25-1.42) |

HR, hazard ratio; CI, confidence interval; CMD, cardiometabolic disease; FCMD, first cardiometabolic disease; CMM, cardiometabolic multimorbidity.

Cardiometabolic diseases include ischemic heart disease, stroke, and type 2 diabetes. Cardiometabolic multimorbidity is defined as occurring at least two of the above-mentioned diseases.

Multivariable models were stratified by age in the 5-year interval, region area, and adjusted for sex, education, marital status, family history of CMM, as appropriate.

Sensitivity analyses included: (1) calculating the entering date of the prior state using different time intervals (0.5-day, 0.5-year, 1-year, 3-year, 5-year) for participants who entered different states on the same day; (2) additionally adjusting for hypertension at baseline; (3) including participants who had previously diagnosed heart disease, stroke, or diabetes, and assigning them to FCMD or CMM state according to their CMD status at baseline; (4) excluding the events occurring in the first two years follow-up; (5) In addition to the predefined five transitions, adding another transition from the baseline directly to CMM.

High-risk lifestyle factors were defined as follows: current smoking or having stopped because of illness; daily drinking ≥30g/d of pure alcohol or having stopped drinking habit; non-daily eating of vegetables, fruits, and eggs, and eating red meat daily or less than weekly; engaging in a sex- and age-specific lower half of total physical activity; having BMI <18.5 or ≥28.0 kg/m^2^ or having waist circumference ≥90cm (men) / 85cm (women).

Figure S1. Timeline of the CKB study


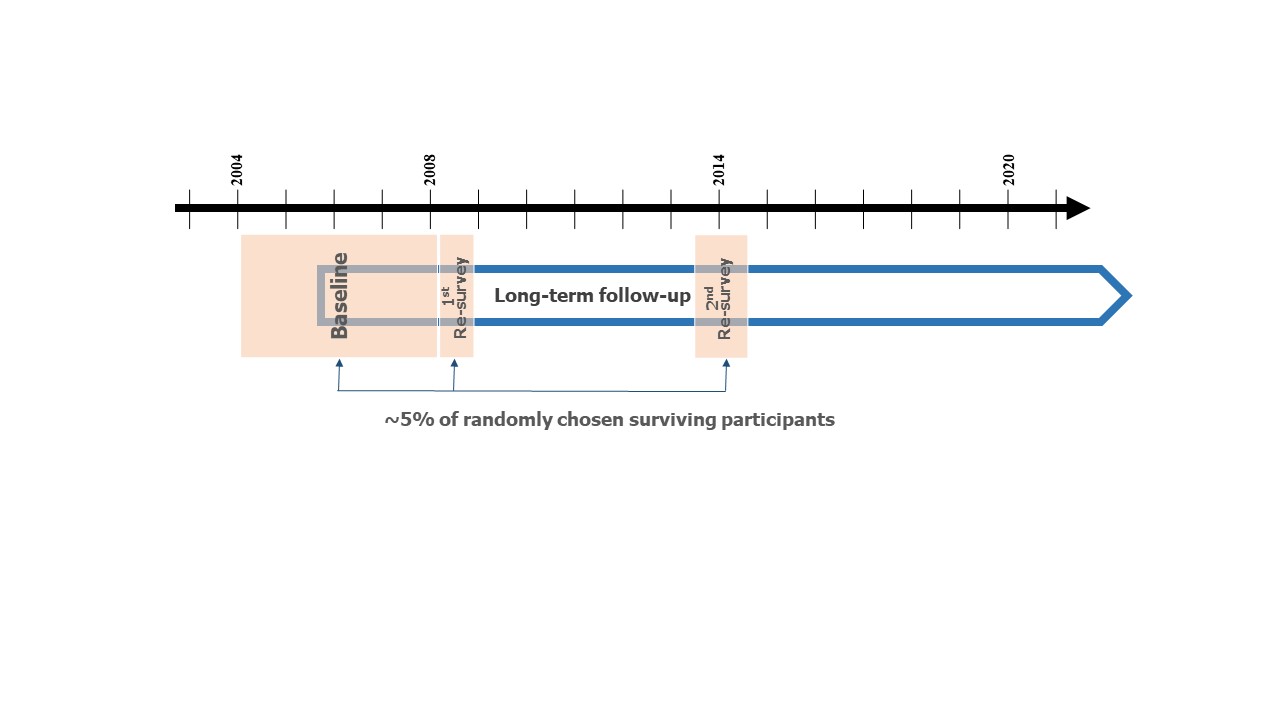


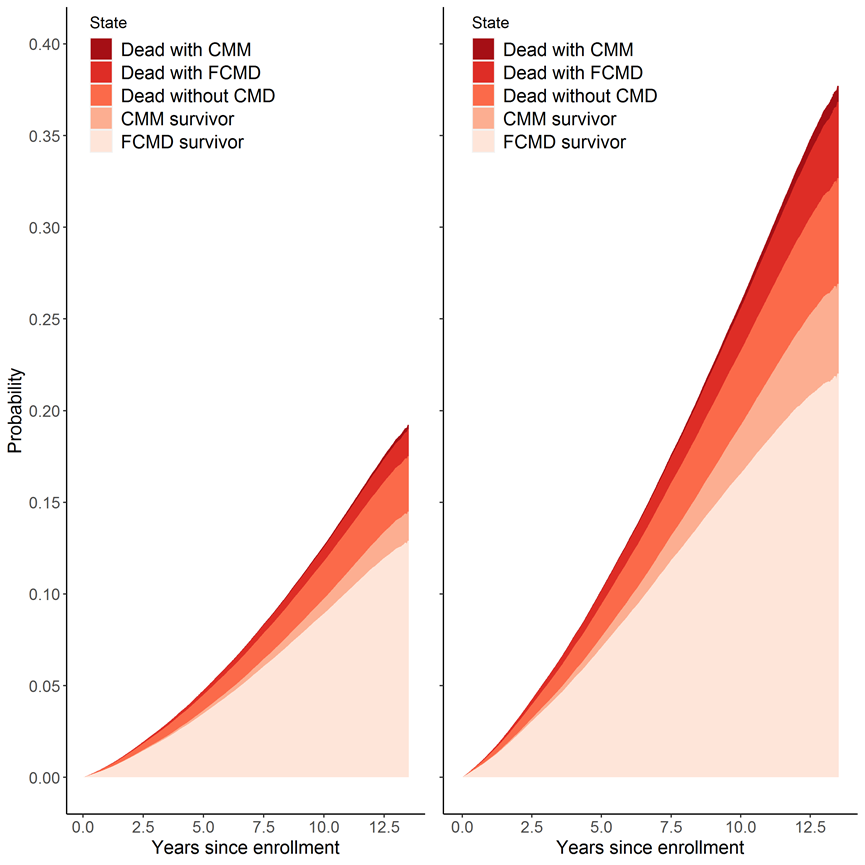


Figure S2. Transition probabilities from baseline (free of cardiometabolic diseases [CMD]) to incident first cardiometabolic disease (FCMD), cardiometabolic multimorbidity (CMM), and death with or without CMD and CMM.

Cardiometabolic diseases include ischemic heart disease, stroke, and type 2 diabetes. Cardiometabolic multimorbidity is defined as occurring at least two of the above-mentioned diseases.

Computed for participants with 0-1 high-risk lifestyle factors (left) and 5 high-risk lifestyle factors (right). All covariates were set to the average level of the CKB population in the present analysis.

Figure S3. Subgroup analysis of associations of the number of high-risk lifestyles factors with morbidity transitions among 461 047 participants


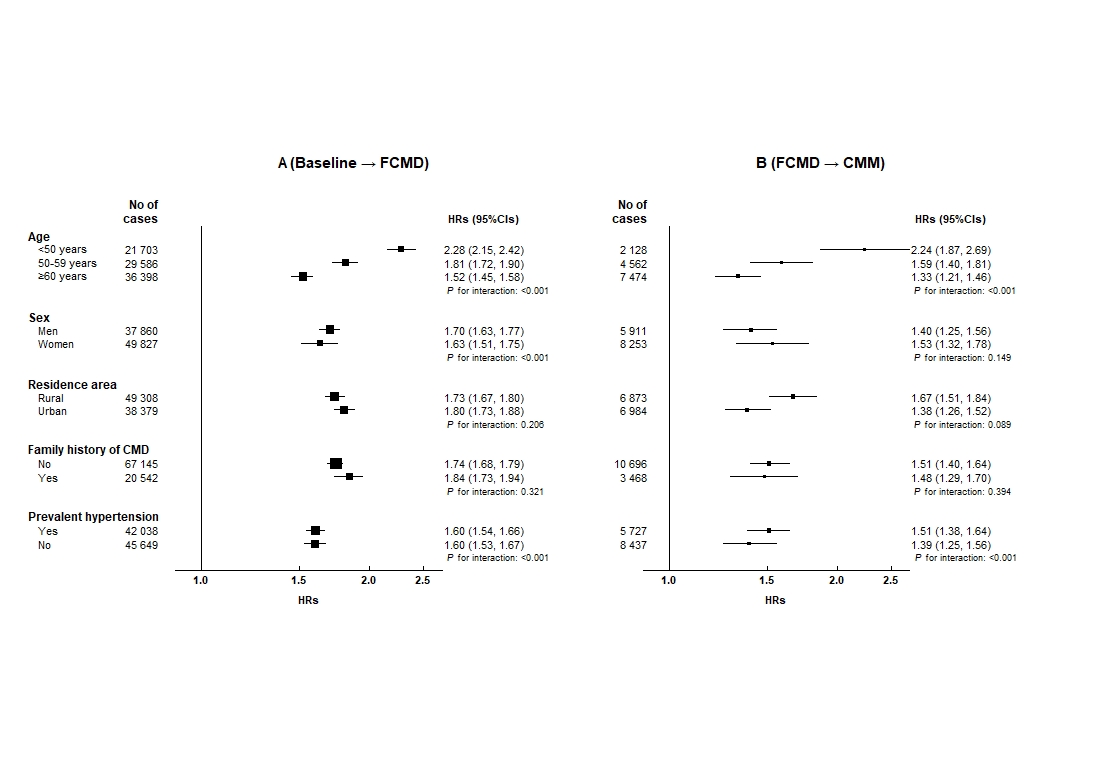


HR, hazard ratio; CI, confidence interval; FCMD, first cardiometabolic disease; CMM, cardiometabolic multimorbidity.

Cardiometabolic diseases include ischemic heart disease, stroke, and type 2 diabetes. Cardiometabolic multimorbidity is defined as occurring at least two of the above-mentioned diseases.

Multivariable models were stratified by age in the 5-year interval, study area, and transition, and adjusted for sex, education, marital status, menopause status (women only), parental family history of CMM, as appropriate.

HRs (95% CIs) were for having 4-5 high-risk lifestyle factors versus having 0-1 high-risk lifestyle factors.

High-risk lifestyle factors were defined as follows: current smoking or having stopped because of illness; daily drinking ≥30g/d of pure alcohol or having stopped drinking habit; non-daily eating of vegetables, fruits, and eggs, and eating red meat daily or less than weekly; engaging in a sex- and age-specific lower half of total physical activity; having BMI <18.5 or ≥28.0 kg/m^2^ or having waist circumference ≥90cm (men) / 85cm (women).

Figure S4. Subgroup analysis of associations of the number of high-risk lifestyles factors with mortality transitions among 461 047 participants


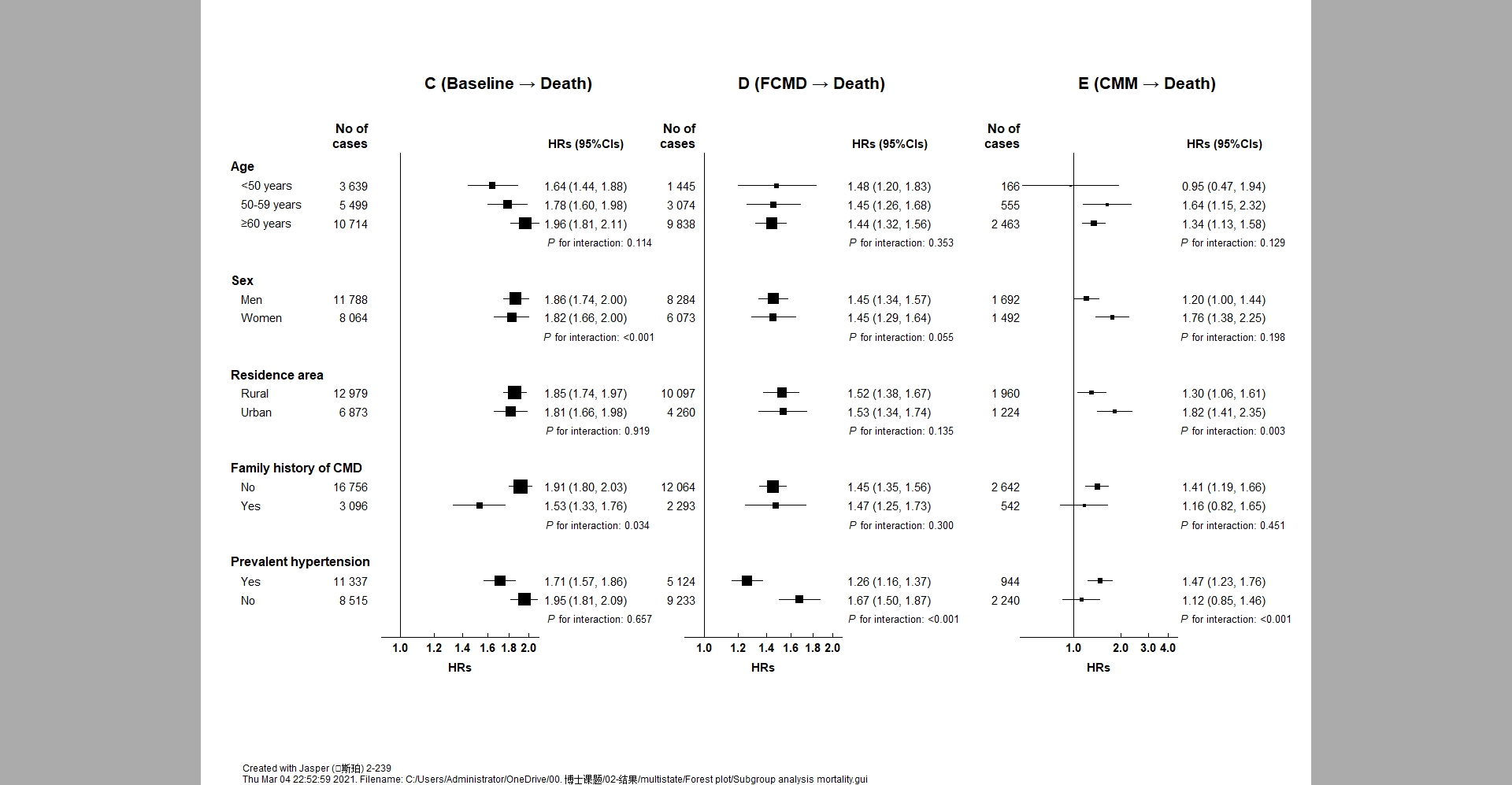


HR, hazard ratio; CI, confidence interval; FCMD, first cardiometabolic disease; CMM, cardiometabolic multimorbidity.

Cardiometabolic diseases include ischemic heart disease, stroke and type 2 diabetes. Cardiometabolic multimorbidity is defined as occurring at least two of the above-mentioned diseases.

Multivariable models were stratified by age in the 5-year interval, study area, and transition, and adjusted for sex, education, marital status, menopause status (women only), parental family history of CMM, as appropriate.

HRs (95% CIs) were for having 4-5 high-risk lifestyle factors versus having 0-1 high-risk lifestyle factors.

High-risk lifestyle factors were defined as follows: current smoking or having stopped because of illness; daily drinking ≥30g/d of pure alcohol or having stopped drinking habit; non-daily eating of vegetables, fruits, and eggs, and eating red meat daily or less than weekly; engaging in a sex- and age-specific lower half of total physical activity; having BMI <18.5 or ≥28.0 kg/m^2^ or having waist circumference ≥90cm (men) / 85cm (women).
